# The close association of Muribaculum and PA (10:0/a-17:0) with the occurrence of pancreatic ductal adenocarcinoma and immunotherapy

**DOI:** 10.3389/fimmu.2024.1505966

**Published:** 2024-11-29

**Authors:** Enzhao Wang, Kuiwu Ren, Xiangyu Wang, Sen Du, Xiang Gao, Wang Niu, Chenyue Guan, Xue Liu, Panpan Wu, Chunlong Liu, Jiangtao Yu, Kun Song

**Affiliations:** ^1^ Department of Hepatobiliary and Pancreatic Surgery, Fuyang People’s Hospital, Fuyang, Anhui, China; ^2^ Department of Hepatobiliary and Pancreatic Surgery, the Affiliated Fuyang Hospital of Bengbu Medical University, Fuyang, Anhui, China; ^3^ Department of Hepatobiliary and Pancreatic Surgery, the Affiliated Fuyang People's Hospital of Anhui Medical University, Fuyang, Anhui, China; ^4^ Graduate School, Wannan Medical College, Wuhu, Anhui, China

**Keywords:** 16S rRNA sequencing, metabolomics, bile, PDAC, immunotherapy

## Abstract

**Background:**

Progress in immunotherapy for pancreatic ductal adenocarcinoma (PDAC) has been slow, yet the relationship between microorganisms and metabolites is crucial to PDAC development. This study compares the biliary microbiota and metabolomic profiles of PDAC patients with those of benign pancreatic disease patients to investigate PDAC pathogenesis and its relationship with immunotherapy.

**Methods:**

A total of 27 patients were recruited, including 15 diagnosed with PDAC and 12 with benign pancreaticobiliary conditions, all of whom underwent surgical treatment. Intraoperative bile samples were collected and analyzed using 16S rRNA sequencing in conjunction with liquid chromatography-mass spectrometry (LC-MS). Multivariate statistical methods and correlation analyzes were employed to assess differences in microbial composition, structure, and function between malignant and benign pancreatic diseases. Additionally, a retrospective analysis was conducted on PDAC patients post-surgery regarding immunotherapy and its correlation with metabolic components.

**Results:**

PDAC patients exhibited a significantly higher abundance of bile microbiota compared to controls, with notable differences in microbiota structure between the two groups (P < 0.05). At the genus level, Muribaculum was markedly enriched in the bile of PDAC patients and was strongly correlated with phosphatidic acid (PA) (10:0/a-17:0). Both of these components, along with the tumor marker CA199, formulated a predictor of PDAC. Furthermore, PA (10:0/a-17:0) demonstrated a strong correlation with PDAC immunotherapy outcomes (Rho: 0.758; P=0.011).

**Conclusion:**

These findings suggest that the biliary microbiota and associated metabolites play a crucial role in the development of PDAC and may serve as potential predictive biomarkers and therapeutic targets for disease management.

## 1 Introduction

Pancreatic cancer (PC), which primarily arises from pancreatic ductal epithelial cells, is an aggressive disease with subtle clinical symptoms, often detected at advanced stages and associated with poor outcomes (1). Global cancer statistics indicate that PC ranks as the sixth most prevalent cancer worldwide, with 511,000 new cases and 467,000 deaths recorded in 2022, marking it as one of the deadliest cancers (2). Hence, timely identification and diagnosis of PC are essential for enhancing patient outcomes and quality of survival.

Recognized risk factors for PC include smoking, adiposity, impaired glucose metabolism, and excessive alcohol consumption (3). Additionally, emerging research suggests that dysregulation of the gut microbiome might be contributing to developing and progressing PC (4). A significant proportion of microorganisms are also being recognized as potential risk factors for pancreatic malignancy (5), further emphasizing the importance of studying the relationship between microbiota and PC.

Traditionally, the pancreas was considered a sterile organ; however, this notion has been increasingly challenged and questioned as research progresses. Although anatomically adjacent to the duodenum, the pancreas remains shielded under normal physiological conditions by the Sphincter of Oddi (SO), which effectively prevents intestinal microbiota from entering the pancreaticobiliary tract, thus making microbial translocation to the pancreas difficult (6). However, when the barrier function is impaired due to factors such as increased intestinal permeability due to inflammation (7, 8), or pathological states such as sphincter of Oddi dysfunction (SOD), the transfer of microorganisms to the bile and pancreatic ducts becomes easier (9). Studies using duodenoscopy have demonstrated that SOD can cause pancreatic bile reflux, which facilitates microbial translocation (10). It has also been proposed that microorganisms may reach the pancreas via the intestinal lymphatic system (11), and recent developments in nanomedicine targeting PC via the intestinal lymphatic pathway further support this hypothesis (12).

Currently, chemotherapy remains the primary treatment for PDAC (13), yielding unsatisfactory results, with the clinical median survival for first-line regimens not exceeding one year (14). While immunotherapy is also a significant treatment modality, the unique immune microenvironment of pancreatic cancer has unfortunately hindered the progress of immunotherapy research for PDAC (15). Recent studies have demonstrated a strong connection between the microbiome and the immune environment of PDAC. A Mendelian randomization analysis identified 20 microorganisms that influence PC through mediators such as Interleukin-6 (IL-6) and CD4 (16). Smruti Pushalkar and colleagues found that microorganisms can inhibit monocyte differentiation through selective Toll-like receptor activation, leading to T-cell inactivation, immunosuppression, and promotion of PC. They also observed that different microbiomes were associated with different stages of malignancy (17). Notably, research has confirmed that Akkermansia and Muribaculum can induce adaptive immune responses, with Muribaculum also promoting pro-inflammatory cytokine production (18, 19). In a study examining the immune efficacy of PD-1 inhibitors in mice with melanoma, significant changes in the abundance of Muribaculum and the Prevotellaceae NK3B31 group were observed (20), further supporting the role of the microbiota in PC development and its modulation of immune cell behavior.

Various methodologies are available for microbiological studies, with 16S rRNA gene profiling being the primary technique to characterize this diverse and distributed microbial community in different human hosts (21). The 16S rRNA gene, a component of the small ribosomal subunit RNA in prokaryotes, contains both conserved and hypervariable regions, the latter reflecting species specificity and phylogenetic relationships, making it an optimal molecular marker for taxonomic classification (22). Analysis of operational taxonomic units (OTUs) or amplicon sequence variants (ASVs) generated from Illumina sequencing allows for comprehensive characterization of microbial community structures within samples (23). For example, Mitsuhashi et al. found that Clostridium spp. was higher plentiful in PC people compared to Individuals in good health using 16S rRNA sequencing, and its abundance was independently associated with poor prognosis in PC tissues (24). Other researchers have also developed a disease diagnostic model for PC progression-free survival by analysing PDAC patients into different groups such as preoperative, postoperative, and progression-free survival (25).

In the mechanisms underlying PC tumorigenesis, various metabolic pathways have been identified, with multiple specific components recognized as potential targets for PC immunotherapy (26). Currently, research focusing on PC metabolites primarily employs metabolomics tools (27). LC-MS combines the advantages of liquid chromatography, including low sample volume, simple sample preparation, low-temperature detection, and efficient separation, with the identification capabilities of mass spectrometry, making it the most widely used analytical technique in metabolomics research (28, 29). For instance, a lipidomic analysis of plasma from 361 PDAC patients identified three distinct metabolic subtypes of PDAC (30). More recently, Hyeonji Kim and colleagues analyzed 12 protein biomarkers in the serum of PDAC patients using LC-MS and developed a predictive model that, when combined with carbohydrate antigen 19-9 (CA19-9), demonstrated improved diagnostic performance (31).

In this study, we integrated 16S rRNA sequencing and LC-MS non-targeted metabolomics to conduct a comprehensive analysis of bile samples from patients with PDAC. Our aim was to assess the influence of microorganisms and metabolites from the biliary microenvironment on PDAC. We sought to provide novel methodologies for the early diagnosis of PDAC in clinical practice while exploring potential targets for immunotherapy.

## 2 Materials and methods

### 2.1 Cohort recruitment and sample collection

This research complied with the ethical standards set forth in the 1975 Declaration of Helsinki and received approval from the Ethics Committee of Fuyang People’s Hospital (approval number: [2024]89). All participants were fully informed about the objectives and procedures of the study, and written consent was obtained from each individual. The study included patients diagnosed with PDAC (Group A, n = 15) or benign pancreaticobiliary diseases, such as intraductal papillary mucinous neoplasms (IPMN) and solid pseudopapillary tumors of the pancreas (SPTP) (Group B, n = 12) All patients underwent surgical intervention and were selected from a cohort spanning January 2022 to June 2023. In total, 27 choledochal bile samples were prospectively collected during surgery — 15 from PDAC patients and 12 from patients with benign diseases. Exclusion criteria included a history of cancer, prior chemotherapy, biliary surgery or stent placement, acute or chronic pancreatitis, pancreatic pseudocysts, pancreatic trauma, and cholangitis, as these conditions could influence the study outcomes.

### 2.2 Specimen collection and preservation

After anesthesia and before any invasive bile duct manipulation, 5 mL of bile was collected from the lower bile ducts using a sterile 5 mL disposable syringe. Samples were promptly transferred into liquid nitrogen and preserved at -80°C in a cryogenic freezer. For analysis, samples were thawed and homogenized at room temperature. Bile samples was dispensed in a sterile biological safety cabinet, aliquoted into sterile anti-adhesion centrifuge tubes using pipettes, and preserved on dry ice for subsequent LC-MS analysis and 16S rRNA sequencing.

### 2.3 16S rRNA sequencing workflow

The commercial kit employed for the extraction of genomic DNA was subjected to analysis using the NanoDrop 2000 (Thermo Fisher Scientific, USA), and the results were corroborated through agarose gel electrophoresis. Subsequently, the sample was subjected to polymerase chain reaction (PCR) amplification after being diluted to a concentration of 1 ng/μL. The 16S rRNA gene was amplified by Takara Ex Taq, with the primers 343F and 798R employed to target the V3-V4 regions. The amplified product was purified using AMPure XP beads (Agencourt) prior to reamplification. Normalisation of the products was conducted with the Qubit dsDNA assay, after which sequencing was performed on Illumina NovaSeq 6000 platform.

Raw FASTQ sequences were subjected to adapter trimming with cutadapt, followed by quality control (QC), denoising, and chimera elimination through DADA2 in QIIME2 (version 2020.11) to generate ASVs. The ASVs were annotated using the Silva database (version 138).

### 2.4 Bile metabolomics analysis

A 200 μL aliquot of each bile specimen was extracted using 400 μL methanol (1:1, v/v), followed by 30 minutes of sonication at 40 kHz and 5°C. Following precipitation at -20°C and centrifugation (13,000 g, 15 min, 4°C), the supernatant was evaporated under a nitrogen stream. For UHPLC-MS/MS analysis, metabolites were dissolved in 100 μL acetonitrile (1:1, v/v), centrifuged, and transferred for analysis.

A pooled QC sample underwent creation through the combination of equivalent parts from each of the samples to ensure system stability and analytical consistency. QC samples underwent identical procedural and analytical processing as that applied to the analytical samples.

The LC-MS analysis was conducted on a Thermo Fisher UHPLC-Q Exactive instrument. A 2 μL aliquot was injected onto an HSS T3 column (100 mm × 2.1 mm i.d., 1.8 μm) and then analyzed via mass spectrometry. The mobile phases used were 0.1% formic acid in water (95:5 v/v) as solvent A, and acetonitrile: isopropanol (47.5:47.5:5, v/v) as solvent B. Mass spectrometry data covering the mass range of 70-1050 m/z were collected using the Thermo UHPLC-Q Exactive mass spectrometer, with the operating mode set to positive and negative electrospray ionization (ESI). Data were acquired in Data -Dependent Acquisition (DDA) mode.

The Progenesis QI software (Waters Corporation, Milford, USA) was employed to process the LC-MS raw data, resulting in the generation of a three-dimensional matrix. Internal standard peaks and noise were removed, and metabolites were annotated using The Majorbio databases, Metlin (https://metlin.scripps.edu/), and Human Metabolome Database (HMDB) (http://www.hmdb.ca/). The Data were normalized, filtered, and log-transformed. Variables with a relative standard deviation (RSD) > 30% in QC samples were excluded.

### 2.5 Statistical analysis

All statistical evaluations were performed using R software (version 4.3.0). Continuous variables were assessed for normality with the Shapiro-Wilk test. Clinical baseline data for normally distributed variables were presented as mean ± standard deviation (SD), while those not meeting normality were expressed as the median and interquartile range (IQR). Group comparisons for continuous variables were conducted using the independent samples t-test. For non-normally distributed variables, comparisons were made using the Mann-Whitney U test. Categorical data were analyzed through Fisher’s exact test.

Microbial diversity was assessed through alpha diversity (Chao1, ACE, Shannon, and Simpson indices) and beta diversity (weighted UniFrac). Non-metric multidimensional scaling (NMDS) and principal coordinates analysis (PCoA) were applied to depict group distinctions. The Wilcoxon statistical test was applied using the R package, and significant differences between groups were identified. Taxonomic abundance variation was assessed via the linear discriminant analysis effect size (LEfSe) approach.

Metabolomic data underwent analysis through the R package ropls, including orthogonal partial least squares discriminant analysis (OPLS-DA) and principal component analysis (PCA), with 7-fold cross-validation to confirm model robustness. The student’s t-test and fold-change analysis identified significantly different metabolites, with Variable Importance in Projection (VIP) > 1 and p < 0.05, leading to the identification of 507 differential metabolites. These metabolites were integrated into metabolic pathways via the Kyoto Encyclopedia of Genes and Genomes (KEGG) database (http://www.genome.jp/kegg/). Enrichment analysis evaluated metabolic pathways, and Fisher’s exact test, along with Python’s scipy.stats package, was used to identify statistically enriched pathways.

Correlation analyzes between microbial taxa, metabolites, and clinical metrics were conducted using Spearman’s rank correlation coefficients. P-values were corrected for multiple testing via the Benjamini-Hochberg method, with an adjusted p-value (q-value) < 0.05 taken as statistically significant. ROC curves were generated using R software, plotting sensitivity against 1-specificity at various threshold levels to assess the predictive capability of significant microorganisms, differential metabolites, and clinical indicators of the disease. The area under the ROC curve (AUC) was calculated to evaluate the diagnostic performance of the models. The standard error (SE) of the AUC was estimated based on the sample size, providing an indication of the variability in the AUC measurement. Additionally, the 95% confidence intervals (CI) for the AUC were estimated using the DeLong method to reflect the robustness of the findings.

## 3 Results

### 3.1 Comparison of clinical baseline data

**Figure 1:**
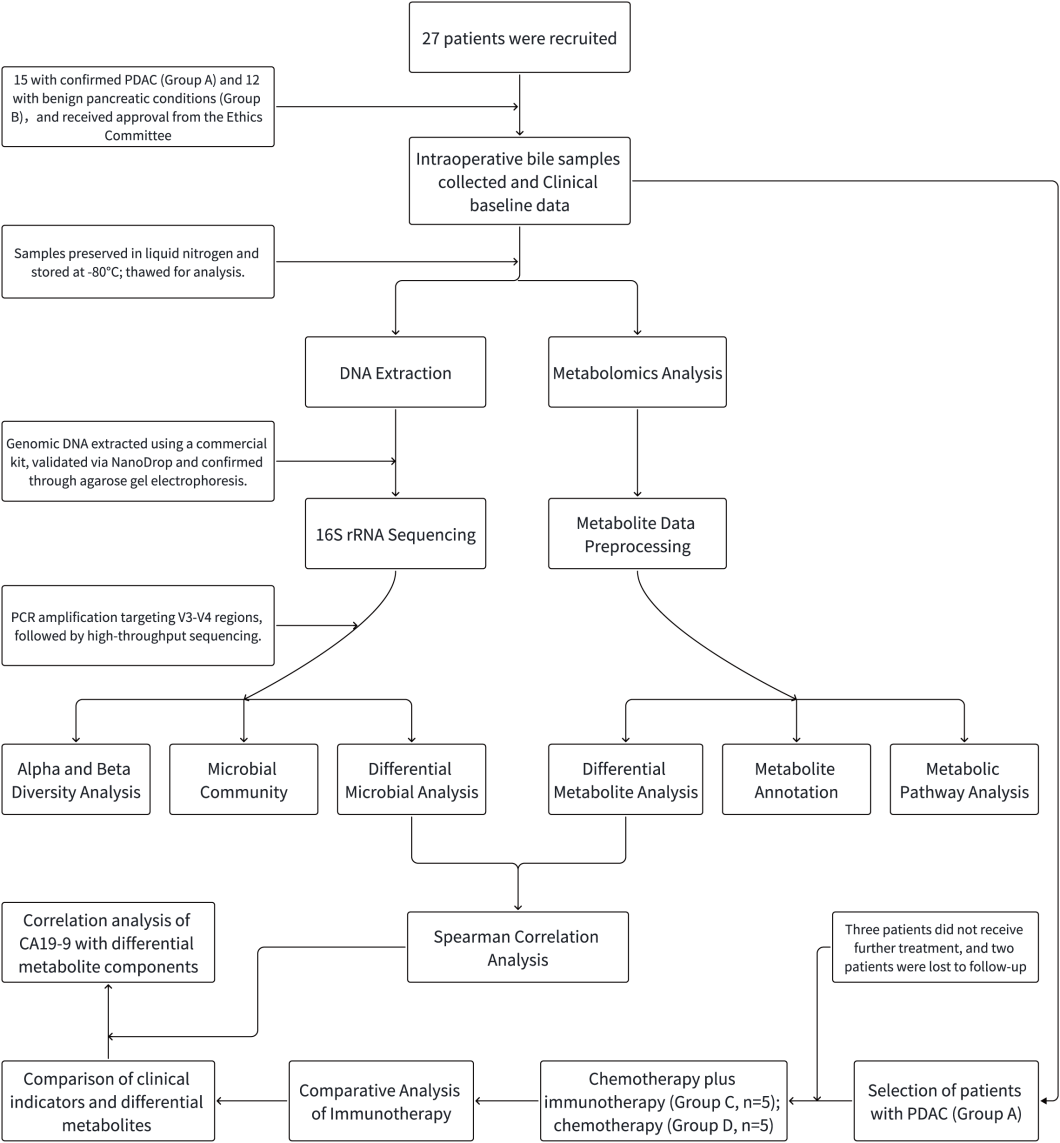
Schematic flowchart of the study design. The flowchart includes patient selection criteria and enrollment status, as well as the processes for 16S rRNA sequencing analysis and LC-MS non-targeted metabolomics. PDAC patients are subsequently assigned to different treatment groups (chemotherapy + immunotherapy, chemotherapy alone). The flowchart concludes with a comparison of clinical indicators and the correlation between CA19-9 levels and specific metabolic components.

This study evaluated the baseline clinical characteristics of 15 individuals diagnosed with PDAC (Group A) and 12 individuals with non-malignant conditions (Group B). The specific process can be referenced in the flowchart (**Figure 1**). The results indicated that Group A had significantly higher levels of total bilirubin (TBIL), direct bilirubin (DBIL), indirect bilirubin (IBIL), and gamma-glutamyl transpeptidase (GGT), which reflect biliary obstruction, compared to Group B. This was expected due to the obstruction of bile excretion. Additionally, a notable difference in CA19-9 concentrations was detected between groups (P<0.05), while no significant differences were found in other clinical indicators (**Table 1**).

**Table 1:** General characteristics and laboratory indicators of patients with PDAC (Group A) and benign pancreaticobiliary diseases (Group B).

Clinical Parameter	A (n=15)	B (n=12)	P value
Age (years)*	68.0 (59.0 ± 72.0)	65.5 (47.0 ± 75.0)	0.494
Gender			
Male, n (%)	11 (73.3)	7 (58.3)	0.448
Female, n (%)	4 (26.7)	5 (41.7)	
BMI (kg/m^2^) **	23.0 ± 3.6	22.0 ± 3.4	0.448
Smoking, n (%)	7 (46.7)	2 (16.7)	0.217
Drinking, n (%)	6 (40.0)	3 (25.0)	0.683
TBIL (umol/L) *	190.1 (69.0 ± 262.9)	19.4 (14.4 ± 90.6)	0.005
DBIL (umol/L) * IBIL (umol/L) * ALT (U/L) *	157.8 (50.4 ± 210.6) 30.8 (15.0 ± 54.9) 131.8 (71.8 ± 257.2)	5.1 (3.9 ± 79.2) 11.4 (9.9 ± 15.0) 50.3 (15.4 ± 173.2)	0.005 0.010 0.157
AST (U/L) *	107.4 (57.0 ± 165.6)	45.2 (18.3 ± 108.1)	0.079
ALP (U/L) *	382.7 (226.0 ± 527.6)	138.8 (99.6 ± 334.8)	0.057
GGT (U/L) *	609.4 (315.7 ± 837.5)	138.7 (14.8 ± 303.4)	0.015
TP (g/dL) **	66.7 ± 5.7	70.5 ± 9.1	0.199
ALB(g/L) **	38.1 ± 3.5	40.2 ± 3.8	0.167
GLU (mmol/L) *	5.4 (4.6 ± 7.5)	5.4 (4.8 ± 6.2)	0.608
Creatinin (mg/dL) **	53.8 ± 10.0	60.8 ± 13.5	0.137
PT(S) **	12.0 ± 0.9	12.7 ± 1.1	0.118
APTT (S) **	28.9 ± 4.0	31.2 ± 5.3	0.204
CEA (ng/mL) *	3.3 (2.8 ± 4.7)	2.9 (1.7 ± 3.6)	0.087
CA19-9 (U/mL) *	166.1 (40.3 ± 1144.6)	18.0 (14.8 ± 52.3)	0.010
WBC(*10^9/L) *	5.9 (4.36 ± 6.6)	6.1 (4.5 ± 9.5)	0.188

ALT, Alanine Aminotransferase; AST, Aspartate Aminotransferase; ALP, Alkaline Phosphatase; TP, Total Protein; ALB, Albumin; GLU, Glucose; PT, Prothrombin Time; APTT, Activated Partial Thromboplastin Time; CEA, Carcinoembryonic Antigen. *The median and IQR; **Mean ± SD; Other data are presented as number (prevalence).

### 3.2 PDAC bile microenvironment exhibits specific microbiological signatures

A total of 27 bile samples from both groups were subjected to high-depth 16S rRNA sequencing, yielding 2,148,739 tags. After processing with DADA2 in QIIME2, 1,541,001 high-quality tags remained, with an average of 57,074 tags per sample, which were clustered into 6,319 ASVs.

**Figure 2:**
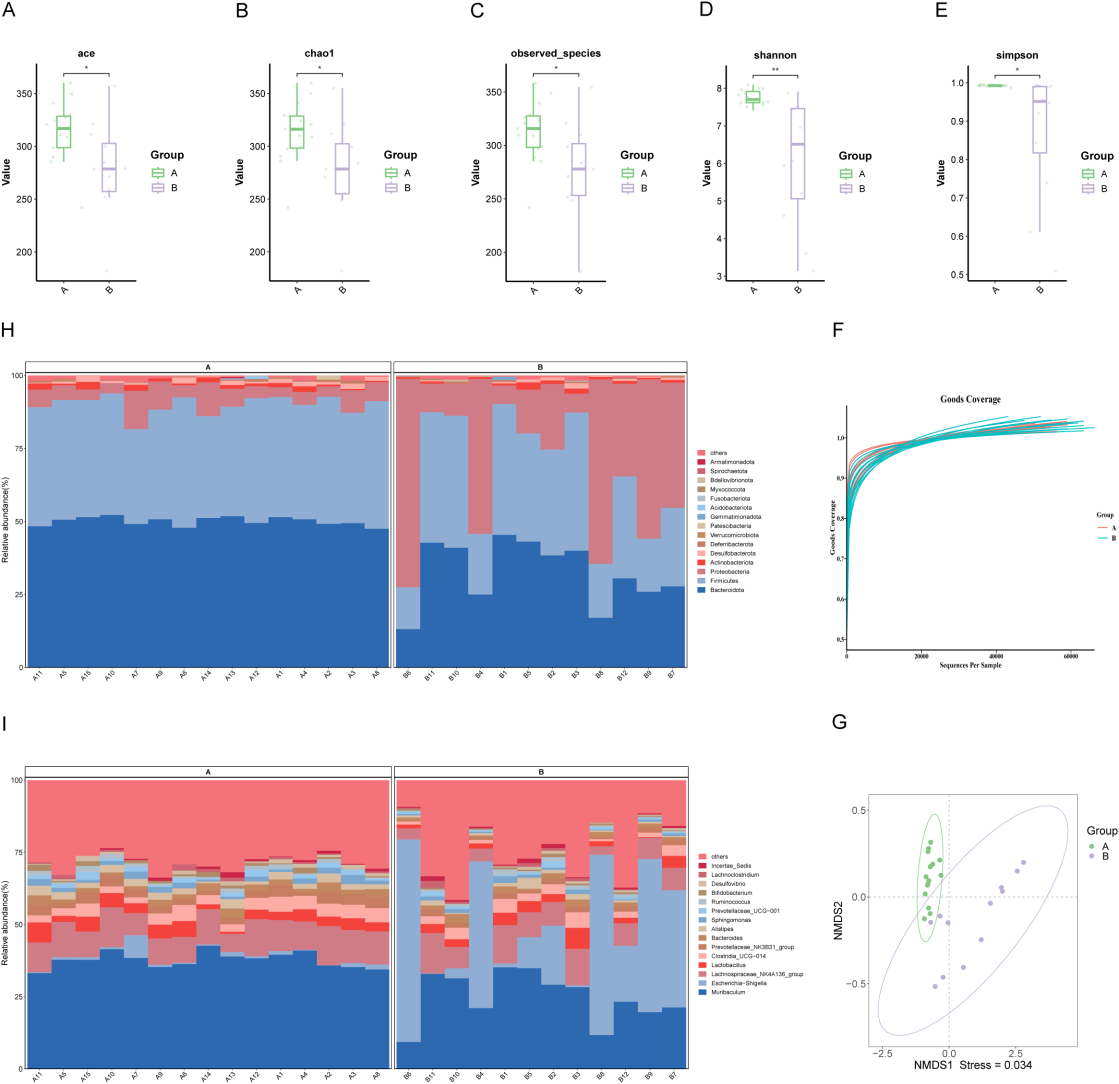
Characterization of biliary microbial community structure in patients with PDAC (Group A) and benign diseases (Group B). **(A–E)** Alpha diversity analysis: Boxplots showing ACE index, Chao1 index, and observed species, which reflect microbial richness, while Shannon and Simpson indices indicate community diversity. **(F)** Species accumulation curve: The curve plateaus as the number of extracted sequences increases, indicating sufficient sequencing depth. **(G)** NMDS: Each point represents a sample, with colors denoting groupings. Closer proximity of points within the same group, coupled with distinct separation between groups, indicates a strong clustering effect. **(H, I)** Composition of microbial communities at the phylum and genus levels in bile samples from groups A and B. “Others” denotes species outside the top-ranked taxa.

The microbial richness and diversity between the groups were compared. The Chao, ACE, and observed species indices were markedly elevated in Group A relative to Group B, indicating that the microbial species in the pancreatic tumor microenvironment were richer than in benign conditions (*P<0.05, **Figures 2A–C**). Additionally, the Shannon index and Simpson index were also significantly different, suggesting distinct microbial diversity between the two groups (*P<0.05; **P<0.01, **Figures 2D, E**). From the Goods coverage curve, it can be seen that the curve rises sharply and then flattens out, which indicates that the sequencing depth is more reasonable and basically covers all the species in the samples (**Figure 2F**).

NMDS and PCoA of weighted UniFrac beta diversity showed significant separation between samples from malignant and benign conditions (Stress: 0.034; ANOSIM R: 0.613, P=0.001, **Figure 2G**; PERMANOVA R²: 0.371, P=0.001, **Supplementary Figure S1**). These findings suggest a clear structural difference between the two microbiotas.

Further analysis of the microbial community structure revealed that at the phylum level, Bacteroidota, Firmicutes, Proteobacteria, Actinobacteriota, Desulfobacterota, and Deferribacterota were dominant, with Bacteroidota and Firmicutes more abundant in PDAC patients than in those with benign disease (**Figure 2H**). At the genus level, Muribaculum, Escherichia-Shigella, Lachnospiraceae_NK4A136_group, Lactobacillus, Clostridia UCG-014, and Prevotellaceae_NK3B31_group were more prevalent in PDAC bile (**Figure 2I**).

**Figure 3:**
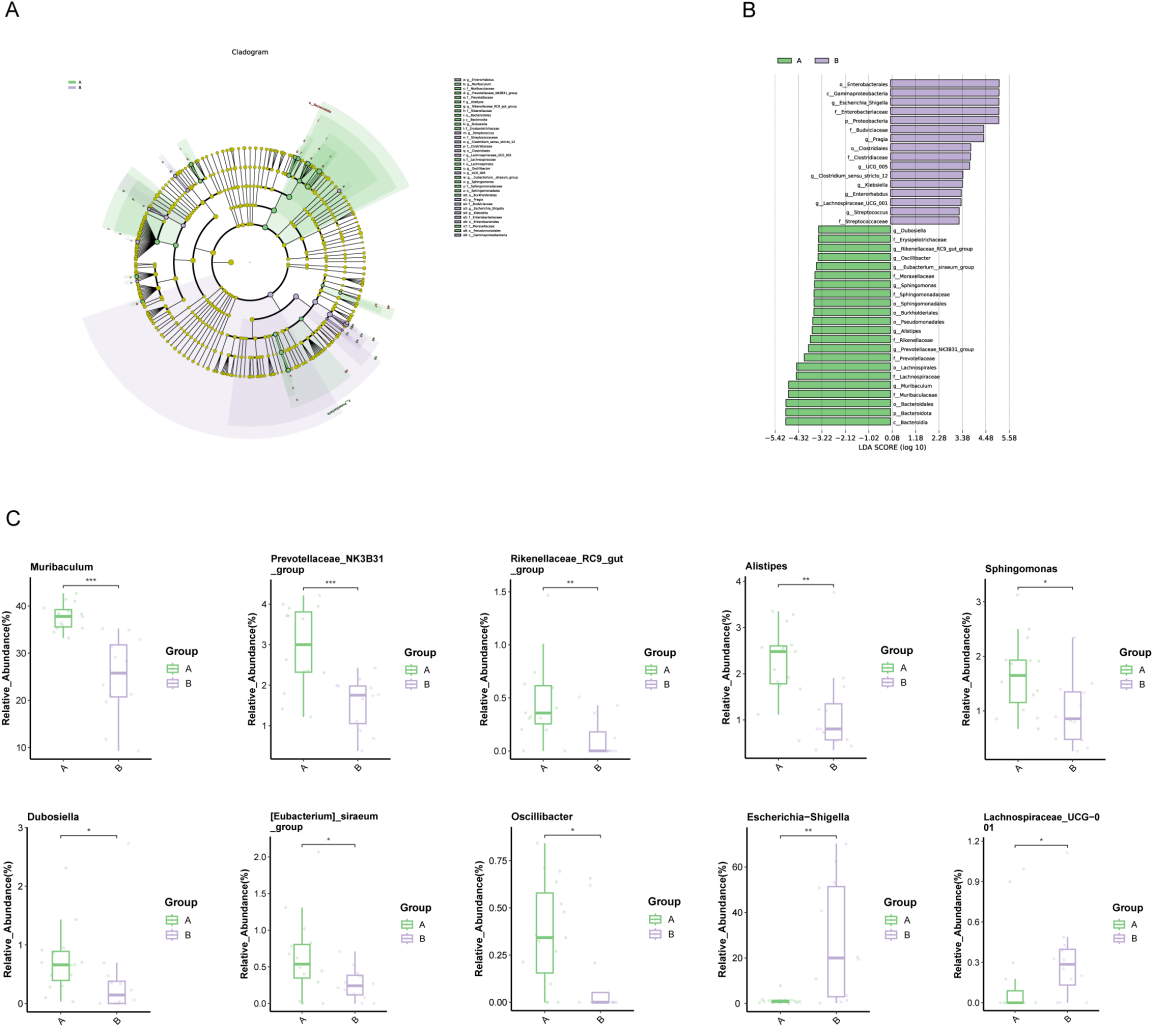
Microbial differences between patients with PDAC (Group A) and benign diseases (Group B). **(A, B)** LEfSe analysis: **(A)** Cladogram depicting the taxonomic structure of differentially abundant species. Light green and light purple indicate significantly enriched species in groups A and B, respectively, with yellow nodes representing taxa without significant differences. The node diameter is proportional to relative abundance, and nodes correspond to taxa at the phylum, class, order, family, and genus levels from inner to outer rings. **(B)** LDA score plot: Light green and light purple bars represent differential species in groups A and B, respectively, with higher relative abundance indicated. **(C)** Boxplot of differential species: Top 10 most abundant species differing between groups (*p < 0.05, **p < 0.01, ***p < 0.001).

LEfSe and linear discriminant analysis (LDA) identified 38 distinct taxa between the groups (**Supplementary Table S1**), with 22 taxa significantly enriched in PDAC patients, including Muribaculum, Prevotellaceae_NK3B31_group, and Rikenellaceae_RC9_gut_group (LDA score < -3.2, P<0.05, **Figures 3A–C**). In contrast, Escherichia-Shigella and Lachnospiraceae_UCG_001 were among the 16 taxa significantly enriched in Group B (LDA score > 3.2, P<0.05).

### 3.3 Pro-cancer metabolic components in the PDAC biliary environment

The metabolic differences between PDAC and benign pancreatic conditions were investigated using LC-MS. In positive ionization mode, 6,096 metabolites were identified, and 6,499 metabolites in negative ion mode. After quality control, 1,954 positive-mode and 1,121 negative-mode metabolites were retained for further analysis. It is evident that the RSD of the processed data is below 0.3, with the cumulative peak occupancy exceeding 70% (**Supplementary Figure S2**).

**Figure 4:**
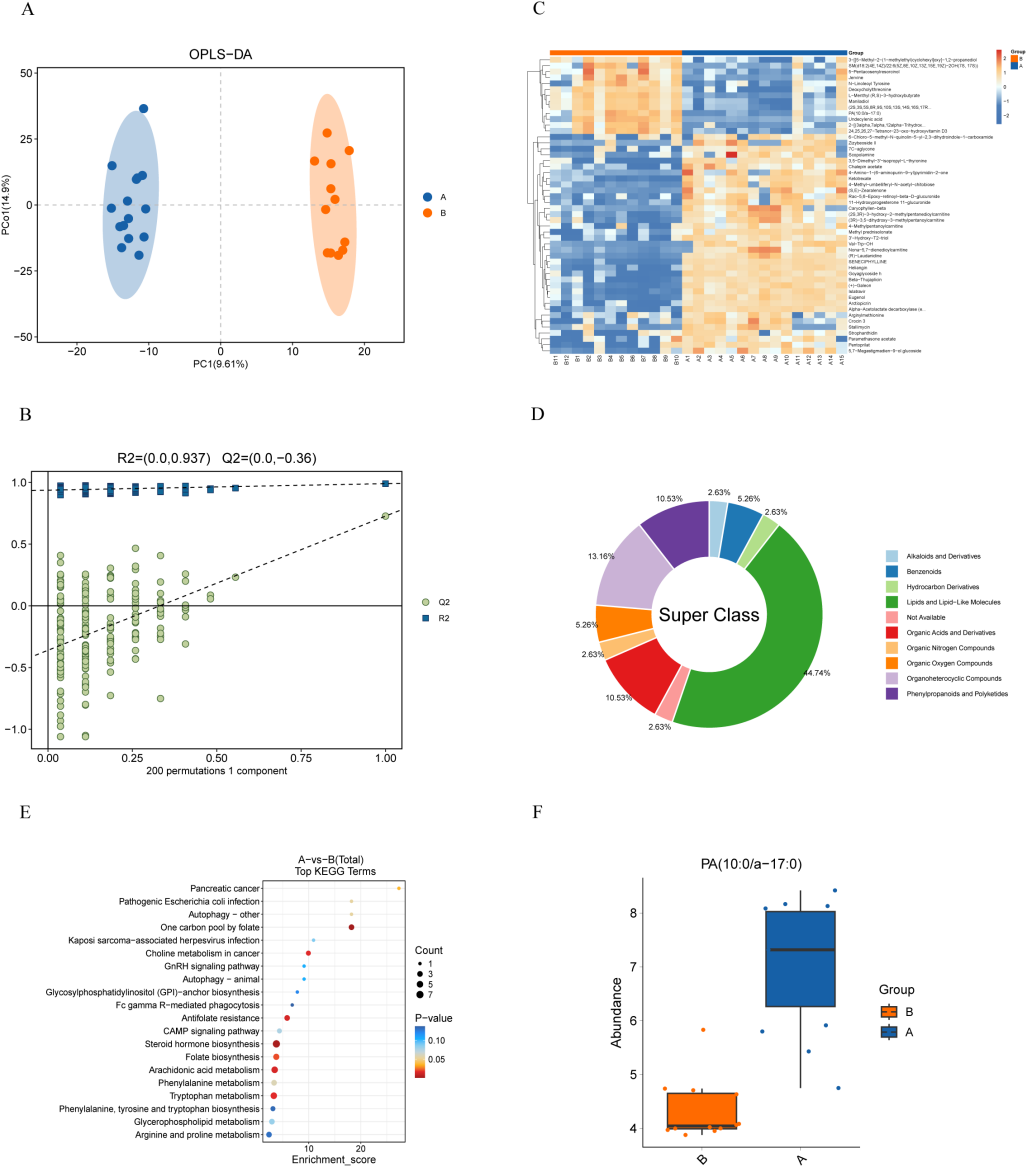
Metabolomic analysis of bile from PDAC patients (Group A) and patients with benign Diseases (Group B). **(A)** OPLS-DA score plot demonstrates distinct metabolite profiles between groups. **(B)** Model validation using a permutation test, with the horizontal axis showing permutation retention and the vertical axis showing R² and Q² values. Dashed lines represent R² and Q² regressions. **(C)** Heatmap of metabolite clustering: Rows and columns represent metabolites and samples, respectively. Colors indicate relative metabolite expression levels, with hierarchical clustering shown for both metabolites (left) and samples (top). **(D)** HMDB compound classification: Differential metabolites were categorized using the HMDB, revealing that lipids and lipid-like molecules dominate at the superclass level. **(E)** KEGG enrichment analysis: The x-axis represents the enrichment score, and the y-axis represents the KEGG pathway. Bubble size reflects the number of metabolites enriched in each pathway, and color indicates p-value significance. **(F)** Boxplot of PA (10:0/a-17:0) distribution: Significantly higher levels of PA (10:0/a-17:0) were found in PDAC patients compared to controls (p < 0.001).

OPLS-DA highlighted notable metabolic differences across the groups, with good predictive ability and no overfitting (Q2(cum)=0.727, R²Y=0.99, **Figures 4A, B**). Metabolites with VIP scores ≥1, identified through the OPLS-DA model and volcano plots (p < 0.05, |log2(FC)| > 0) based on differential quality data, were considered significantly different (**Supplementary Figure S3**). Comparison of these metabolites to the KEGG database resulted in the discovery of 507 unique metabolites. To visualize the relationships between sample groups and the differential expressivity of metabolic compounds, a hierarchical clustering analysis was conducted utilizing all significantly different metabolites and on the top 50 metabolites ranked by VIP score (**Figure 4C**; **Supplementary Table S2**). Querying the HMDB for classification of these top 50 differential metabolites at the superclass level revealed that lipids and lipid-like molecules are the predominant components, accounting for 44.74 percent. (**Figure 4D**).

We classified the differential metabolites into metabolic pathways and conducted enrichment analysis using the KEGG database. The 20 most significantly enriched pathways (smallest p-values) were visualized using a bubble diagram (**Figure 4E**). The analysis identified key pathways, including tryptophan metabolism, arachidonic acid metabolism, folate biosynthesis, steroid hormone biosynthesis, antifolate resistance, choline metabolism in cancer, one-carbon pool by folate, pancreatic cancer, and nine other metabolic pathways (p < 0.05). Among these, PA (10:0/a-17:0) was identified as the primary metabolite in the pancreatic cancer pathway, and its levels were higher in samples from PDAC patients (p < 0.001, **Figure 4F**).

### 3.4 Microbial and metabolic component association analysis

**Figure 5:**
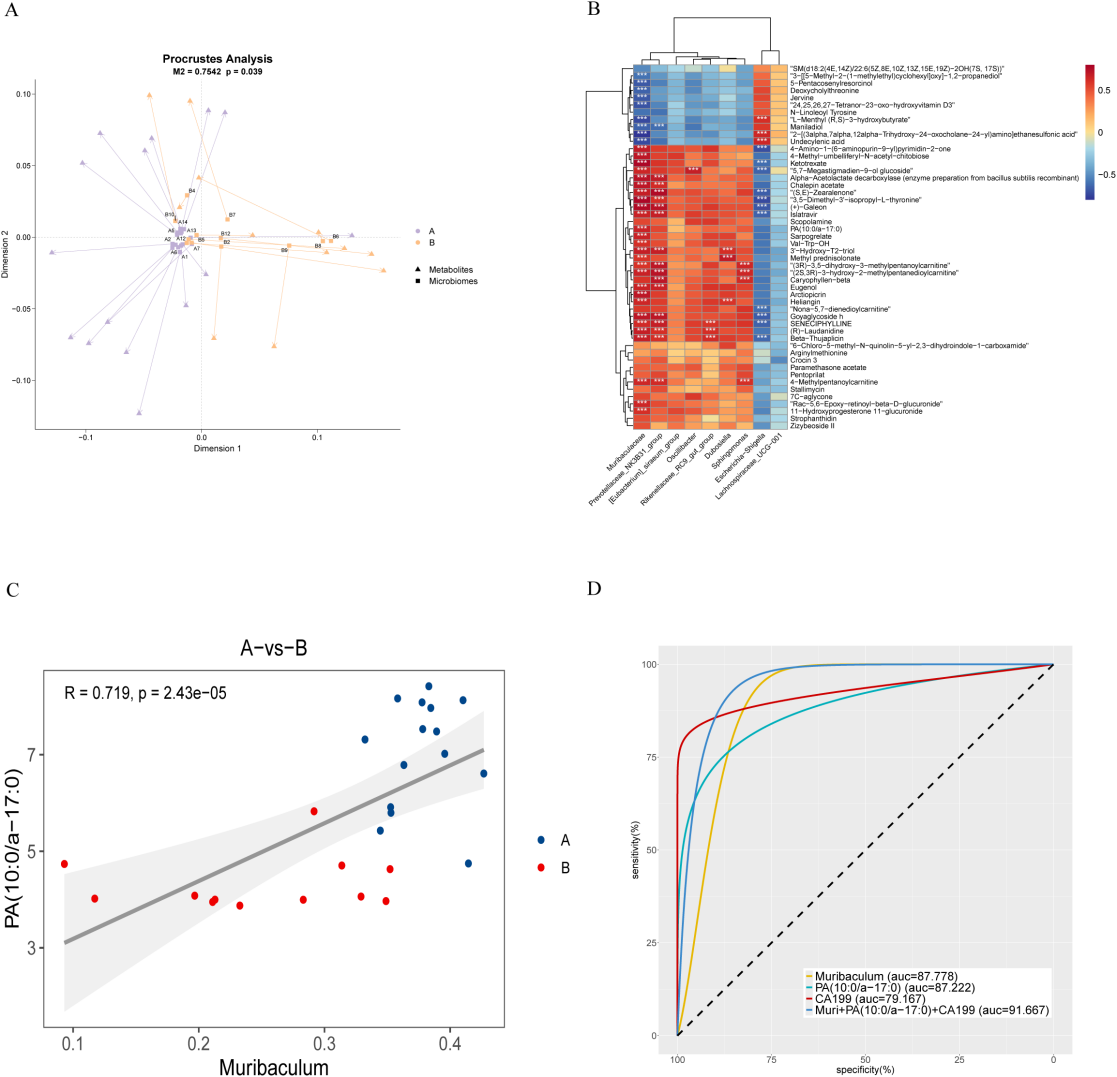
Association analysis of biliary microbiota and metabolites in PDAC patients. **(A)** Procrustes analysis: Arrows show the relationship between microbial (starting point) and metabolite (endpoint) samples. The M² value represents the sum of squared deviations, and the p-value indicates significance, with smaller values suggesting stronger correlation. **(B)** Correlation heatmap: Spearman’s correlation coefficient is used to illustrate relationships between microbiota and metabolites. Red indicates positive correlations, blue negative, with darker shades signifying stronger correlations (*p < 0.05, **p < 0.01, ***p < 0.001). **(C)** Scatterplot of correlation between samples: Colors represent groupings, with linear fit and 95% CI displayed, along with the correlation coefficient (R) and p-value. **(D)** Receiver Operating Characteristic Curve (ROC curve): This curve compares the diagnostic performance of Muribaculum, PA (10:0/a-17:0), CA199, and their combined model. For clarity, we present the AUC as an integer and adjust its decimal point two places to the left for accurate interpretation. A higher value indicates greater diagnostic accuracy.

The association between microbiota and metabolic components was explored by integrating LC-MS data and 16S rRNA sequencing results. The Procrustes test revealed a robust association linking microbial alterations with metabolic changes. (M²=0.7542, P=0.039, **Figure 5A**). Spearman’s correlation analysis further revealed significant associations between specific PDAC-related microorganisms (e.g., Muribaculum, Prevotellaceae_NK3B31_group) and metabolites such as PA (10:0/a-17:0) (**Figure 5B**; **Supplementary Table S3**). Scatter plot analysis demonstrated a high positive association (R=0.719, P<0.0001) linking Muribaculum with PA (10:0/a-17:0) (**Figure 5C**). The combination of Muribaculum, PA (10:0/a-17:0), and CA19-9 showed improved diagnostic efficacy for pancreatic malignancy, yielding an AUC of 0.917 (P<0.001; 95%CI: 0.805-1), outperforming individual biomarkers (**Figure 5D**; **Supplementary Table S4**).

### 3.5 Association between postoperative immunotherapy for PDAC and significant metabolites

Subsequently, we conducted a comparative analysis of 15 PDAC patients based on their receipt of postoperative immunotherapy. Regrettably, 3 patients did not undergo further adjuvant treatment at our institution, and 2 patients were lost to follow-up, resulting in incomplete clinical data. Consequently, these patients were excluded from the current comparative cohort. The remaining patients were stratified into two groups: Group C (n=5), who received gemcitabine, nab-paclitaxel, and camrelizumab, and Group D (n=5), who received gemcitabine and nab-paclitaxel alone. According to the patient’s overall condition, standard chemotherapy and immunotherapy were initiated 4-8 weeks postoperatively. At the third follow-up visit following the second round of adjuvant therapy, CA19-9 levels were re-evaluated and compared to baseline measurements from the initial visit to determine the extent of reduction. Furthermore, additional clinical parameters were assessed to provide a comprehensive analysis (**Table 2**). Unfortunately, no significant differences were found in these indicators between Groups C and D. However, box plots illustrated that patients receiving combined chemotherapy and immunotherapy experienced a greater reduction in CA19-9 levels compared to those treated with chemotherapy alone (**Supplementary Figure S4**).

**Table 2:** Clinical indicators of specific metabolites before surgery and after chemotherapy or immunotherapy in groups C (Chemotherapy + Immunotherapy) and D (Chemotherapy).

Clinical Parameter	C (n=5)	D (n=5)	P value
WBC(*10^9/L) *	3.7 (3.5 ± 6.1)	4.4 (3.6 ± 4.8)	0.916
ALP (U/L) **	109.2 ± 27.8	148.6 ± 61.3	0.227
CEA (ng/mL) **	2.6 ± 1.30	3.7 ± 1.9	0.303
CA19-9 (U/mL) *	536.4 (66.6 ± 3733.9)	411.3 (213.0 ± 3426.4)	0.917
Ratio**	0.234 ± 0.319	0.008 ± 0.194	0.214
PA (10:0/a-17:0) **	8.065 ± 0.326	7.034 ± 0.846	0.035

*The median and IQR; **Mean ± SD; The Ratio is calculated as the difference between the pre-immunotherapy and post-immunotherapy CA19-9 levels, divided by the pre-immunotherapy CA19-9 value.

Furthermore, we observed that the preoperative levels of PA (10:0/a-17:0) were significantly higher in Group C compared to Group D (P=0.035). Spearman correlation analysis further revealed a strong association between the magnitude of CA19-9 reduction and PA (10:0/a-17:0) levels (Rho: 0.758; P=0.011;**Supplementary Table S5**). These findings imply a potential relationship between PA (10:0/a-17:0) and the effectiveness of immunotherapy in PDAC, suggesting that it may represent a promising immunotherapeutic target; however, additional evidence is required to substantiate this association.

## 4 Discussion

In recent years, high-throughput genomic techniques have been increasingly utilized in human microbiome research. These studies have shown that microorganisms and metabolic component are essential contributors to the development and advancement of numerous diseases (32). Among these, the microbiota significantly contributes to the pathogenesis of PDAC, influencing tumorigenesis, immune responses, and treatment outcomes (33, 34). Given the current slow progress in PC research, investigating the impact of microorganisms on tumor development and potential immune targets is crucial. This study compared the biliary microenvironment and metabolic profiles between patients with PDAC and those with benign conditions, in order to identify specific microorganisms and differential metabolites, and to explore their associations with the efficacy of immunotherapy. Prior to this study, no comprehensive analysis had been performed on microbial and metabolite profiles in PDAC by obtaining bile samples during surgery via an abdominal approach.

In this study, 16S rRNA sequencing of bile collected from individuals with PC and benign lesions revealed that microbial diversity and abundance were significantly higher in the PC cohort compared to the benign group. This result is consistent with the findings of Erick Riquelme et al., who found that microbial α-diversity were notably higher in long-term survivors (LTS) than in short-term survivors (STS) of PC, with α-diversity serving as a potential predictor of PC resectability (35). Additionally, Beta analysis identified substantial variation in the overall microbiological community structure between both cohorts, mirroring trends observed in previous related studies (25).

Previous research has shown that microbial dysbiosis may be closely linked to immune evasion, inflammatory responses, and tumor microenvironment remodeling during PC development (36). These microbial communities may also influence the efficacy of cancer immunotherapies, such as anti-PD-1 therapeutics (37). Certain microorganisms, such as Porphyromonas gingivalis (P. gingivalis), have been strongly associated with an increased prevalence of PC, as demonstrated in meta-analysis (38). Other bacteria promote PC progression by stimulating neutrophilic chemokine and elastase secretion (39). In our analysis, Bacteroidota, Muribaculum, and the Prevotellaceae NK3B31 group showed significant enrichment in PDAC, consistent with prior studies characterizing the microbial community of PC (40–42). This also reflects that the distinct compositions of microbial communities within the biliary microenvironment may influence the prognosis of PDAC in the context of immunotherapy. Understanding these relationships could provide a foundation for tailoring PDAC immunotherapeutic strategies based on individual microbial profiles.

In addition to the microbiota, previous studies have identified a robust correlation between various metabolic profiles and the development of PC (43). Anatomically, the bile duct is connected to the pancreas, and the composition of bile has a substantial impact on pancreatic diseases. For instance, microRNAs in bile have been shown to enhance the diagnostic accuracy of PC (44). Additionally, volatile organic compounds in bile, as in as acetaldehyde and acetone, can aid in PC diagnosis (45). In this study, we employed metabolomic analysis to investigate the metabolic profiles of bile samples and found significant differences between PDAC and benign lesions. In PDAC patients, the top 50 distinct metabolic components were predominantly lipids and lipid-like components. Some of these metabolites can be hydrolyzed into fatty acids and glycerol, with gut microbes influencing pancreatic immunity through short-chain fatty acids (46).

Metabolic pathway analysis identified PA (10:0/a-17:0) as a key metabolite involved in several pathways related to pancreatic diseases. In the pancreatic cancer pathway, for example, mutation-activated KRAS signaling through RalGDS involves PA via phospholipase D1/2 (PLD1/2). PA (10:0/a-17:0) is a downstream metabolite of PLD1, which plays a more significant role in pancreatic development than previously recognized. PLD1 has been shown to inhibit TGF-beta signaling, blocking G(1) cell cycle progression and thereby promoting the anaplastic growth of tumor cells (47). Moreover, the small scaffold protein can block procedural death of Ins-1E pancreatic β-cells through PLD1 (48). Phospholipases also contribute to advancing renal cell carcinoma by facilitating angiogenesis (49). Additionally, PA (10:0/a-17:0) participates in the phospholipase D signaling pathway, contributing to glycosylphosphatidylinositol (GPI) cleavage, which produces glypicans. These glypicans act as insulin mediators, supporting glucose metabolism to supply energy to PC cells (50). Thus, PA (10:0/a-17:0) is intricately associated with the initiation and progression of PC.

Spearman correlation and regression analyzes revealed a significant positive correlation between Muribaculum and PA (10:0/a-17:0). Further multiple regression analysis showed that combining these microorganisms and their metabolic components with the PDAC biomarker CA19-9 enhanced diagnostic accuracy, outperforming CA19-9 alone (AUC: 0.917vs.0.792).

Phospholipids are widely present in the cell membranes of eukaryotic cells and have a significant impact on immunotherapy for pancreatic tumors (51). They influence membrane fluidity and signal transduction, regulating the activity and migration of immune cells, while tumors can evade immune surveillance by altering the composition of membrane phospholipids, thereby impairing the function of T cells and other immune cells (52, 53). The correlation analysis between postoperative chemotherapy combined with immunotherapy and PA (10:0/a-17:0) also suggests the potential application of this phospholipid component in PDAC immunotherapy, indicating its possibility as a therapeutic target.

In previous pancreatic microbiome research, samples were primarily collected via the gastrointestinal tract, such as fecal samples, which may not accurately represent the pancreatic microenvironment. Alternatively, bile was extracted using endoscopic retrograde cholangiopancreatography (ERCP) from a duodenal approach, but contamination by intestinal microorganisms remains a challenge. In this study, bile was collected directly from the biliary tract using sterile surgical techniques, combined with metabolomic analysis, providing a more accurate reflection of the pancreatic microenvironment. Additionally, we prospectively divided PDAC patients into two groups based on whether they received immunotherapy after surgery, revealing a potential connection between specific metabolic components and immunotherapy. However, it is important to acknowledge the existence of several limitations that must be taken into account. First, the sample size was small, and the study was single-center, limiting the generalizability of the findings. Second, although associations between microbiota alterations, metabolites, and PDAC were identified, the underlying molecular mechanisms remain unclear and require further investigation. Future research should increase the sample size and focus on elucidating the molecular pathways through which microbiota and metabolites contribute to PDAC development, potentially offering new insights into pancreatic cancer prevention and treatment.

## 5 Conclusion

In this study, 16S rRNA sequencing combined with untargeted metabolomics revealed a complex interplay between biliary microbiota and metabolites in the pathogenesis of PDAC. Significant differences were identified in the composition and metabolomic profiles within the biliary microbial communities between PDAC patients and those with benign diseases. Notably, the correlation between Muribaculum and the biliary metabolite PA (10:0/a-17:0), along with associated metabolic pathways, suggests a potential role in PDAC progression. These findings highlight the potential of specific microbial taxa and metabolites as biomarkers for the early detection and diagnosis of PDAC.

## Data Availability

The datasets presented in this study can be found in online repositories. The names of the repository/repositories and accession number(s) can be found below: https://www.ncbi.nlm.nih.gov/, PRJNA1159707 https://ngdc.cncb.ac.cn/, PRJCA030052.
