# Supplementary material for: The close association of Muribaculum and PA (10:0/a-17:0) with the occurrence of pancreatic ductal adenocarcinoma and immunotherapy
Source: Front Immunol. 2024 Nov 29;15:1505966. doi: 10.3389/fimmu.2024.1505966 (PMC11638228; doi:10.3389/fimmu.2024.1505966)
Supplement: Supplementary file 1 [file DataSheet1.docx]

Supplementary Material


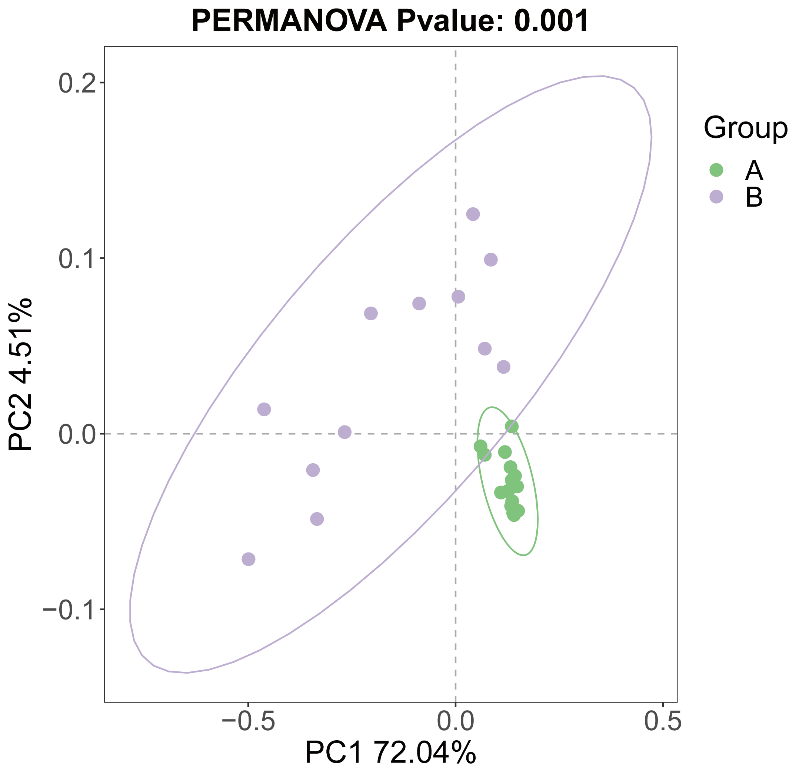


**Figure S1.** PCoA of weighted UniFrac analysis: Each point represents a sample, with colors denoting groupings. Closer proximity within the same group and significant separation between groups suggest effective grouping.


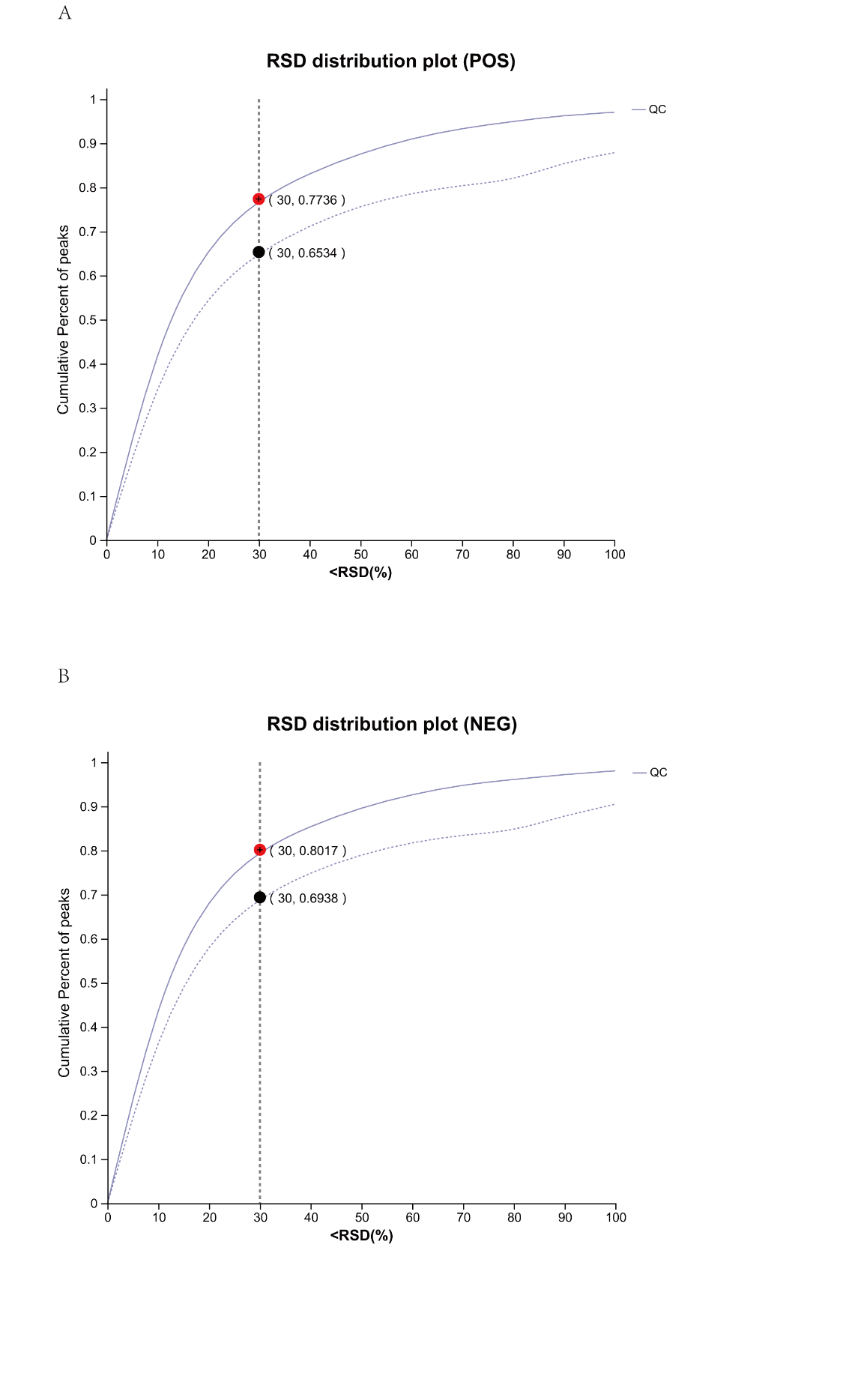


**Figure S2.** QC sample evaluation plots: (A) Cation mode; (B) Anion mode. The x-axis represents the RSD (%) value (standard deviation/mean), while the y-axis shows the cumulative proportion of ion peaks. RSD < 0.3 and cumulative peaks > 70% indicate acceptable data quality. Dashed lines represent pre-treatment, and solid lines indicate post-treatment results.


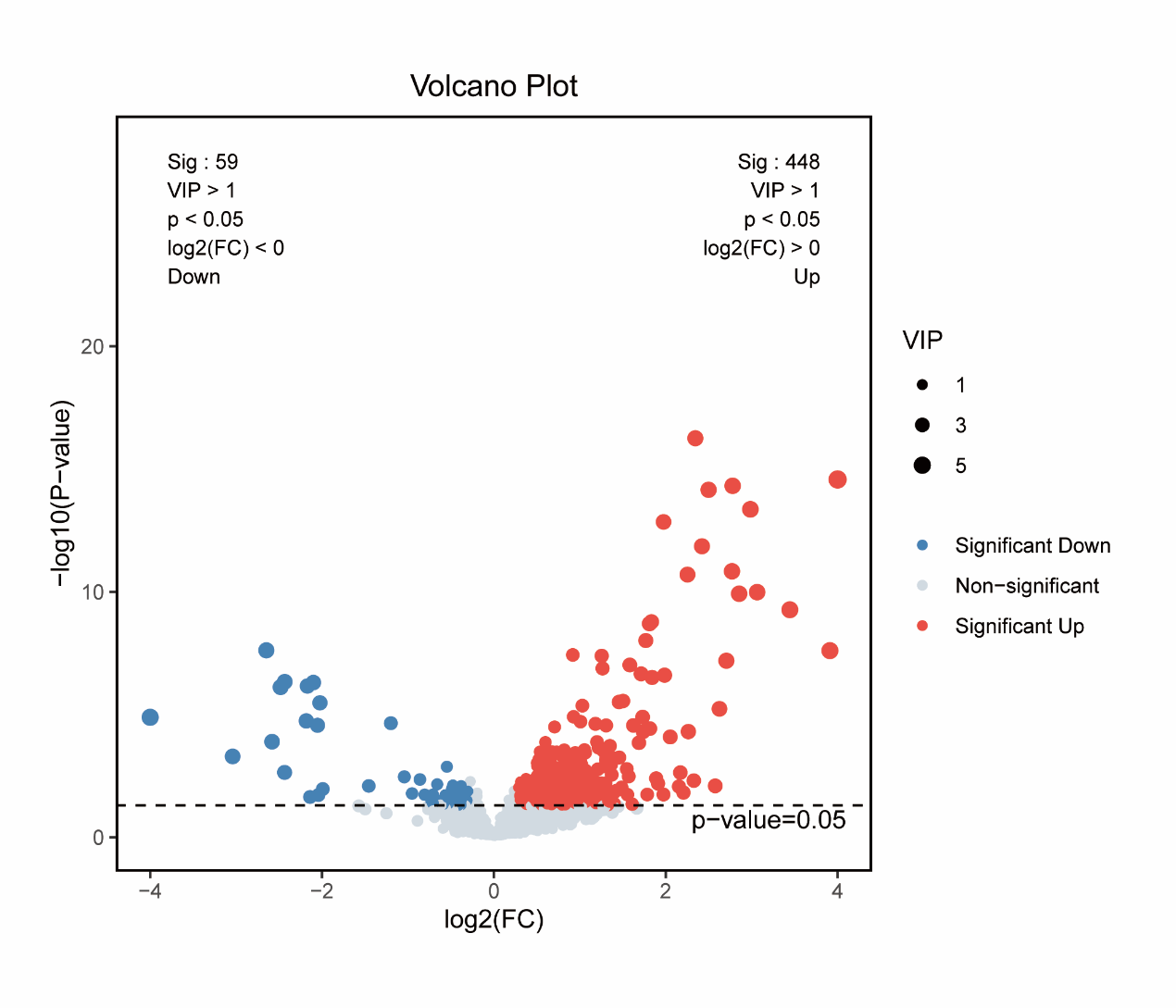


**Figure S3.** Volcano plot of differential metabolites: The x-axis represents the fold change in metabolite expression between the two groups, and the y-axis represents the significance of the expression differences. Each point represents a specific metabolite, with point size corresponding to the VIP value. Red points indicate significantly upregulated metabolites, blue points significantly downregulated, and grey points represent non-significant differences. Points further to the left, right, or top indicate more significant expression differences.


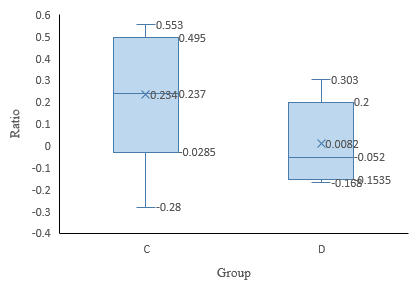


**Figure S4.** Box plots illustrating the magnitude of change in CA19-9 levels before and after treatment in Groups C and D. Each box represents the interquartile range, with the median indicated by a horizontal line and the mean marked by a cross. Whiskers extend to the minimum and maximum values.
