# Supplementary material for: The close association of Muribaculum and PA (10:0/a-17:0) with the occurrence of pancreatic ductal adenocarcinoma and immunotherapy
Source: Front Immunol. 2024 Nov 29;15:1505966. doi: 10.3389/fimmu.2024.1505966 (PMC11638228; doi:10.3389/fimmu.2024.1505966)
Supplement: Supplementary file 2 [file DataSheet2.docx]

Supplementary Material

**Table S1.** Differential microorganisms identified by LEfSe analysis in groups A and B

| Group | Biomarker | Logarithm value | LDA_value | p-value |
| --- | --- | --- | --- | --- |
| A | k__Bacteria.p__Firmicutes.c__Clostridia.o__Oscillospirales.f__Ruminococcaceae.g___Eubacterium__siraeum_group | 3.801454 | 3.520325 | 0.031534 |
| A | k__Bacteria.p__Bacteroidota.c__Bacteroidia | 5.699783 | 4.968971 | 1.13E-05 |
| A | k__Bacteria.p__Bacteroidota.c__Bacteroidia.o__Bacteroidales.f__Rikenellaceae | 4.432424 | 3.873682 | 0.001791 |
| A | k__Bacteria.p__Bacteroidota.c__Bacteroidia.o__Bacteroidales.f__Muribaculaceae.g__Muribaculum | 5.577002 | 4.840202 | 2.71E-05 |
| A | k__Bacteria.p__Bacteroidota.c__Bacteroidia.o__Bacteroidales.f__Rikenellaceae.g__Rikenellaceae_RC9_gut_group | 3.663006 | 3.567127 | 0.009221 |
| A | k__Bacteria.p__Proteobacteria.c__Alphaproteobacteria.o__Sphingomonadales | 4.22836 | 3.569801 | 0.007281 |
| A | k__Bacteria.p__Bacteroidota.c__Bacteroidia.o__Bacteroidales.f__Prevotellaceae | 4.712488 | 4.05626 | 0.000305 |
| A | k__Bacteria.p__Proteobacteria.c__Gammaproteobacteria.o__Pseudomonadales | 2.802212 | 3.565309 | 0.013227 |
| A | k__Bacteria.p__Firmicutes.c__Clostridia.o__Lachnospirales | 5.378682 | 4.532295 | 0.024796 |
| A | k__Bacteria.p__Bacteroidota.c__Bacteroidia.o__Bacteroidales.f__Muribaculaceae | 5.580029 | 4.845498 | 2.71E-05 |
| A | k__Bacteria.p__Firmicutes.c__Bacilli.o__Erysipelotrichales.f__Erysipelotrichaceae | 3.930666 | 3.396334 | 0.031691 |
| A | k__Bacteria.p__Firmicutes.c__Clostridia.o__Oscillospirales.f__Oscillospiraceae.g__Oscillibacter | 3.56306 | 3.445197 | 0.011464 |
| A | k__Bacteria.p__Bacteroidota.c__Bacteroidia.o__Bacteroidales.f__Rikenellaceae.g__Alistipes | 4.348662 | 3.780326 | 0.003991 |
| A | k__Bacteria.p__Firmicutes.c__Clostridia.o__Lachnospirales.f__Lachnospiraceae | 5.378178 | 4.537019 | 0.024796 |
| A | k__Bacteria.p__Firmicutes.c__Bacilli.o__Erysipelotrichales.f__Erysipelotrichaceae.g__Dubosiella | 3.860026 | 3.389285 | 0.011047 |
| A | k__Bacteria.p__Bacteroidota | 5.699783 | 4.968121 | 1.13E-05 |
| A | k__Bacteria.p__Bacteroidota.c__Bacteroidia.o__Bacteroidales.f__Prevotellaceae.g__Prevotellaceae_NK3B31_group | 4.469195 | 3.850358 | 0.00128 |
| A | k__Bacteria.p__Proteobacteria.c__Alphaproteobacteria.o__Sphingomonadales.f__Sphingomonadaceae.g__Sphingomonas | 4.21908 | 3.549419 | 0.012827 |
| A | k__Bacteria.p__Proteobacteria.c__Gammaproteobacteria.o__Burkholderiales | 4.018338 | 3.550351 | 0.016805 |
| A | k__Bacteria.p__Proteobacteria.c__Gammaproteobacteria.o__Pseudomonadales.f__Moraxellaceae | 2.77567 | 3.595087 | 0.025179 |
| A | k__Bacteria.p__Bacteroidota.c__Bacteroidia.o__Bacteroidales | 5.694536 | 4.962913 | 1.13E-05 |
| A | k__Bacteria.p__Proteobacteria.c__Alphaproteobacteria.o__Sphingomonadales.f__Sphingomonadaceae | 4.22836 | 3.569801 | 0.007281 |
| B | k__Bacteria.p__Firmicutes.c__Bacilli.o__Lactobacillales.f__Streptococcaceae | 3.353958 | 3.160772 | 0.027272 |
| B | k__Bacteria.p__Proteobacteria | 5.509578 | 5.156645 | 0.000209 |
| B | k__Bacteria.p__Firmicutes.c__Clostridia.o__Clostridiales.f__Clostridiaceae | 4.163449 | 3.81056 | 0.020161 |
| B | k__Bacteria.p__Proteobacteria.c__Gammaproteobacteria.o__Enterobacterales.f__Enterobacteriaceae.g__Escherichia_Shigella | 5.443704 | 5.162426 | 0.002916 |
| B | k__Bacteria.p__Proteobacteria.c__Gammaproteobacteria.o__Enterobacterales.f__Enterobacteriaceae.g__Klebsiella | 3.494502 | 3.625445 | 0.032065 |
| B | k__Bacteria.p__Proteobacteria.c__Gammaproteobacteria.o__Enterobacterales.f__Budviciaceae | 1.415993 | 4.315261 | 0.04428 |
| B | k__Bacteria.p__Proteobacteria.c__Gammaproteobacteria.o__Enterobacterales.f__Enterobacteriaceae | 5.455058 | 5.162969 | 0.00076 |
| B | k__Bacteria.p__Firmicutes.c__Clostridia.o__Clostridiales | 4.173067 | 3.817279 | 0.004309 |
| B | k__Bacteria.p__Firmicutes.c__Clostridia.o__Clostridiales.f__Clostridiaceae.g__Clostridium_sensu_stricto_12 | 2.953819 | 3.503613 | 0.04428 |
| B | k__Bacteria.p__Actinobacteriota.c__Coriobacteriia.o__Coriobacteriales.f__Eggerthellaceae.g__Enterorhabdus | 3.340672 | 3.342225 | 0.034428 |
| B | k__Bacteria.p__Firmicutes.c__Clostridia.o__Lachnospirales.f__Lachnospiraceae.g__Lachnospiraceae_UCG_001 | 3.487212 | 3.383305 | 0.029095 |
| B | k__Bacteria.p__Proteobacteria.c__Gammaproteobacteria.o__Enterobacterales | 5.463699 | 5.167958 | 0.000141 |
| B | k__Bacteria.p__Proteobacteria.c__Gammaproteobacteria.o__Enterobacterales.f__Budviciaceae.g__Pragia | 1.415993 | 4.392762 | 0.04428 |
| B | k__Bacteria.p__Firmicutes.c__Clostridia.o__Oscillospirales.f__Oscillospiraceae.g__UCG_005 | 2.602879 | 3.708275 | 0.04428 |
| B | k__Bacteria.p__Firmicutes.c__Bacilli.o__Lactobacillales.f__Streptococcaceae.g__Streptococcus | 3.353958 | 3.159783 | 0.027272 |
| B | k__Bacteria.p__Proteobacteria.c__Gammaproteobacteria | 5.486332 | 5.167583 | 0.000253 |

**Table S2.** Summary of significantly different metabolites in VIP top50

| Metabolites | VIP | Regulation | Ion mode | p-value |
| --- | --- | --- | --- | --- |
| SENECIPHYLLINE | 6.001947 | Up | pos | 2.64E-15 |
| Deoxycholylthreonine | 5.028481 | Down | pos | 1.29E-05 |
| 3,5-Dimethyl-3'-isopropyl-L-thyronine | 4.919281 | Up | pos | 2.48E-08 |
| Arctiopicrin | 4.88047 | Up | pos | 5.36E-10 |
| Alpha-Acetolactate decarboxylase (enzyme preparation from bacillus subtilis recombinant) | 4.635713 | Up | neg | 4.34E-14 |
| Val-Trp-OH | 4.492953 | Up | pos | 1.03E-10 |
| Heliangin | 4.464809 | Up | pos | 4.79E-15 |
| Beta-Thujaplicin | 4.443146 | Up | pos | 1.19E-10 |
| Goyaglycoside h | 4.417189 | Up | pos | 1.45E-11 |
| (+)-Galeon | 4.296392 | Up | pos | 6.96E-15 |
| PA(10:0/a-17:0) | 4.184909 | Up | pos | 6.38E-08 |
| L-Menthyl (R,S)-3-hydroxybutyrate | 4.158382 | Down | pos | 2.41E-08 |
| (R)-Laudanidine | 4.158318 | Up | pos | 5.53E-17 |
| Ketotrexate | 4.121405 | Up | neg | 1.39E-12 |
| Maniladiol | 3.996438 | Down | pos | 7.63E-07 |
| Islatravir | 3.991219 | Up | pos | 1.98E-11 |
| 2-[(3alpha,7alpha,12alpha-Trihydroxy-24-oxocholane-24-yl)amino]ethanesulfonic acid | 3.885489 | Down | pos | 4.61E-07 |
| SM(d18:2(4E,14Z)/22:6(5Z,8E,10Z,13Z,15E,19Z)-2OH(7S, 17S)) | 3.861588 | Down | pos | 0.000506 |
| Rac-5,6-Epoxy-retinoyl-beta-D-glucuronide | 3.861078 | Up | neg | 5.82E-06 |
| 3-[[5-Methyl-2-(1-methylethyl)cyclohexyl]oxy]-1,2-propanediol | 3.810431 | Down | pos | 0.000127 |
| Eugenol | 3.785046 | Up | pos | 1.41E-13 |
| (2S,3S,5S,8R,9S,10S,13S,14S,16S,17R)-17-Acetyloxy-10,13-dimethyl-2-morpholin-4-yl-16-(1-prop-2-enylpyrrolidin-1-ium-1-yl)-2,3,4,5,6,7,8,9,11,12,14,15,16,17-tetradecahydro-1H-cyclopenta[a]phenanthren-3-olate | 3.68162 | Down | pos | 4.94E-07 |
| 4-Methylpentanoylcarnitine | 3.59392 | Up | pos | 4.93E-05 |
| Undecylenic acid | 3.590277 | Down | pos | 6.86E-07 |
| 5-Pentacosenylresorcinol | 3.585406 | Down | pos | 1.81E-05 |
| (2S,3R)-3-hydroxy-2-methylpentanedioylcarnitine | 3.570367 | Up | pos | 1.97E-09 |
| 24,25,26,27-Tetranor-23-oxo-hydroxyvitamin D3 | 3.484403 | Down | pos | 3.32E-06 |
| 4-Methyl-umbelliferyl-N-acetyl-chitobiose | 3.457855 | Up | neg | 1.66E-09 |
| Chalepin acetate | 3.442825 | Up | pos | 2.48E-07 |
| (S,E)-Zearalenone | 3.399276 | Up | pos | 3.06E-07 |
| Jervine | 3.376322 | Down | pos | 2.72E-05 |
| 11-Hydroxyprogesterone 11-glucuronide | 3.341672 | Up | neg | 9.61E-09 |
| N-Linoleoyl Tyrosine | 3.322277 | Down | pos | 0.002269 |
| Methyl prednisolonate | 3.265259 | Up | pos | 2.19E-07 |
| Crocin 3 | 3.263694 | Up | pos | 8.14E-05 |
| 6-Chloro-5-methyl-N-quinolin-5-yl-2,3-dihydroindole-1-carboxamide | 3.196624 | Up | neg | 0.007999 |
| 3'-Hydroxy-T2-triol | 3.162713 | Up | pos | 9.52E-08 |
| 4-Amino-1-(6-aminopurin-9-yl)pyrimidin-2-one | 3.114435 | Up | pos | 1.26E-05 |
| Pentoprilat | 3.104369 | Up | pos | 4.98E-05 |
| Caryophyllen-beta | 3.091203 | Up | pos | 2.76E-05 |
| 7C-aglycone | 3.083273 | Up | pos | 3.72E-05 |
| Paramethasone acetate | 3.029547 | Up | pos | 0.002308 |
| (3R)-3,5-dihydroxy-3-methylpentanoylcarnitine | 3.023407 | Up | pos | 3.07E-06 |
| Zizybeoside II | 2.985102 | Up | neg | 0.004876 |
| Scopolamine | 2.976593 | Up | pos | 0.00014 |
| 5,7-Megastigmadien-9-ol glucoside | 2.947058 | Up | pos | 2.79E-06 |
| Arginylmethionine | 2.829966 | Up | pos | 0.01497 |
| Nona-5,7-dienedioylcarnitine | 2.823204 | Up | pos | 4.05E-08 |
| Stallimycin | 2.794158 | Up | pos | 0.006414 |
| Strophanthidin | 2.75432 | Up | pos | 0.003973 |

**Table S3.** Table of parameters for Spearman's correlation analysis

| Metabolites | Microbiomes | rho | P value | relation |
| --- | --- | --- | --- | --- |
| "2-[(3alpha,7alpha,12alpha-Trihydroxy-24-oxocholane-24-yl)amino]ethanesulfonic acid" | Muribaculaceae | -0.78522364 | 1.2306E-06 | negtive |
| Ketotrexate | Muribaculaceae | 0.78308656 | 1.3753E-06 | positive |
| "3,5-Dimethyl-3'-isopropyl-L-thyronine" | Muribaculaceae | 0.77331706 | 2.2521E-06 | positive |
| "(S,E)-Zearalenone" | Prevotellaceae_NK3B31_group | 0.76007326 | 8.1405E-06 | positive |
| (+)-Galeon | Muribaculaceae | 0.75591514 | 5.1208E-06 | positive |
| "3,5-Dimethyl-3'-isopropyl-L-thyronine" | Escherichia-Shigella | -0.73504274 | 2.1467E-05 | negtive |
| "(2S,3R)-3-hydroxy-2-methylpentanedioylcarnitine" | Prevotellaceae_NK3B31_group | 0.72710623 | 2.8882E-05 | positive |
| (+)-Galeon | Prevotellaceae_NK3B31_group | 0.72588523 | 3.0209E-05 | positive |
| Alpha-Acetolactate decarboxylase (enzyme preparation from bacillus subtilis recombinant) | Muribaculaceae | 0.72111129 | 2.1994E-05 | positive |
| "L-Menthyl (R,S)-3-hydroxybutyrate" | Muribaculaceae | -0.7205007 | 2.252E-05 | negtive |
| Undecylenic acid | Muribaculaceae | -0.7205007 | 2.252E-05 | negtive |
| Goyaglycoside h | Prevotellaceae_NK3B31_group | 0.71916972 | 3.8539E-05 | positive |
| "(3R)-3,5-dihydroxy-3-methylpentanoylcarnitine" | Prevotellaceae_NK3B31_group | 0.71916972 | 3.8539E-05 | positive |
| 4-Amino-1-(6-aminopurin-9-yl)pyrimidin-2-one | Escherichia-Shigella | -0.71855922 | 3.939E-05 | negtive |
| 4-Methyl-umbelliferyl-N-acetyl-chitobiose | Muribaculaceae | 0.71561594 | 2.715E-05 | positive |
| Caryophyllen-beta | Prevotellaceae_NK3B31_group | 0.71489621 | 4.4853E-05 | positive |
| Arctiopicrin | Muribaculaceae | 0.71256298 | 3.0458E-05 | positive |
| Goyaglycoside h | Muribaculaceae | 0.7098153 | 3.3736E-05 | positive |
| Chalepin acetate | Muribaculaceae | 0.70737293 | 3.691E-05 | positive |
| Methyl prednisolonate | Dubosiella | 0.7013154 | 4.5955E-05 | positive |
| 4-Amino-1-(6-aminopurin-9-yl)pyrimidin-2-one | Muribaculaceae | 0.69760343 | 5.2424E-05 | positive |
| Alpha-Acetolactate decarboxylase (enzyme preparation from bacillus subtilis recombinant) | Prevotellaceae_NK3B31_group | 0.6959707 | 8.5165E-05 | positive |
| "24,25,26,27-Tetranor-23-oxo-hydroxyvitamin D3" | Muribaculaceae | -0.68691804 | 7.5786E-05 | negtive |
| SENECIPHYLLINE | Prevotellaceae_NK3B31_group | 0.68681319 | 0.00011415 | positive |
| (R)-Laudanidine | Muribaculaceae | 0.68386507 | 8.3967E-05 | positive |
| 3'-Hydroxy-T2-triol | Muribaculaceae | 0.68355977 | 8.4827E-05 | positive |
| (+)-Galeon | Escherichia-Shigella | -0.68315018 | 0.00012796 | negtive |
| 5-Pentacosenylresorcinol | Muribaculaceae | -0.68142269 | 9.1066E-05 | negtive |
| Beta-Thujaplicin | Muribaculaceae | 0.67959091 | 9.6733E-05 | positive |
| Jervine | Muribaculaceae | -0.67806443 | 0.00010169 | negtive |
| 3'-Hydroxy-T2-triol | Prevotellaceae_NK3B31_group | 0.67399267 | 0.00016901 | positive |
| "5,7-Megastigmadien-9-ol glucoside" | Oscillibacter | 0.67385901 | 0.00011653 | positive |
| "(S,E)-Zearalenone" | Muribaculaceae | 0.67348497 | 0.00011794 | positive |
| Islatravir | Prevotellaceae_NK3B31_group | 0.67338217 | 0.00017211 | positive |
| Islatravir | Muribaculaceae | 0.66707374 | 0.00014453 | positive |
| PA(10:0/a-17:0) | Muribaculaceae | 0.66279958 | 0.00016508 | positive |
| "(2S,3R)-3-hydroxy-2-methylpentanedioylcarnitine" | Muribaculaceae | 0.66249428 | 0.00016664 | positive |
| "(S,E)-Zearalenone" | Escherichia-Shigella | -0.65934066 | 0.00025857 | negtive |
| Eugenol | Muribaculaceae | 0.65913602 | 0.00018469 | positive |
| Eugenol | Prevotellaceae_NK3B31_group | 0.65811966 | 0.00026761 | positive |
| Goyaglycoside h | Escherichia-Shigella | -0.65689866 | 0.00027693 | negtive |
| Sarpogrelate | Muribaculaceae | 0.65638835 | 0.00020072 | positive |
| "5,7-Megastigmadien-9-ol glucoside" | Muribaculaceae | 0.65425127 | 0.00021402 | positive |
| Undecylenic acid | Escherichia-Shigella | 0.65262515 | 0.0003118 | positive |
| "(3R)-3,5-dihydroxy-3-methylpentanoylcarnitine" | Sphingomonas | 0.65201465 | 0.00031708 | positive |
| Deoxycholylthreonine | Muribaculaceae | -0.65150359 | 0.00023226 | negtive |
| "5,7-Megastigmadien-9-ol glucoside" | Escherichia-Shigella | -0.64835165 | 0.00035041 | negtive |
| "(2S,3R)-3-hydroxy-2-methylpentanedioylcarnitine" | Sphingomonas | 0.64652015 | 0.00036819 | positive |
| Maniladiol | Muribaculaceae | -0.64539766 | 0.00027775 | negtive |
| "2-[(3alpha,7alpha,12alpha-Trihydroxy-24-oxocholane-24-yl)amino]ethanesulfonic acid" | Escherichia-Shigella | 0.64529915 | 0.00038047 | positive |
| Islatravir | Escherichia-Shigella | -0.64346764 | 0.00039956 | negtive |
| SENECIPHYLLINE | Escherichia-Shigella | -0.64224664 | 0.00041275 | negtive |
| Heliangin | Muribaculaceae | 0.6408182 | 0.00031682 | positive |
| "Rac-5,6-Epoxy-retinoyl-beta-D-glucuronide" | Muribaculaceae | 0.63959702 | 0.00032803 | positive |
| 4-Methylpentanoylcarnitine | Muribaculaceae | 0.63929172 | 0.00033088 | positive |
| SENECIPHYLLINE | Muribaculaceae | 0.63745994 | 0.00034847 | positive |
| Chalepin acetate | Prevotellaceae_NK3B31_group | 0.63247863 | 0.00053251 | positive |
| Beta-Thujaplicin | Escherichia-Shigella | -0.63064713 | 0.00055803 | negtive |
| 4-Methylpentanoylcarnitine | Prevotellaceae_NK3B31_group | 0.62820513 | 0.00059371 | positive |
| Beta-Thujaplicin | Rikenellaceae_RC9_gut_group | 0.62710768 | 0.00046411 | positive |
| Caryophyllen-beta | Sphingomonas | 0.62271062 | 0.00068128 | positive |
| (R)-Laudanidine | Prevotellaceae_NK3B31_group | 0.62026862 | 0.00072366 | positive |
| 3'-Hydroxy-T2-triol | Dubosiella | 0.61907746 | 0.00057566 | positive |
| "Nona-5,7-dienedioylcarnitine" | Escherichia-Shigella | -0.61721612 | 0.00077982 | negtive |
| (R)-Laudanidine | Rikenellaceae_RC9_gut_group | 0.61457806 | 0.00064786 | positive |
| 11-Hydroxyprogesterone 11-glucuronide | Muribaculaceae | 0.61364678 | 0.00066376 | positive |
| Val-Trp-OH | Muribaculaceae | 0.61303618 | 0.00067436 | positive |
| "3,5-Dimethyl-3'-isopropyl-L-thyronine" | Prevotellaceae_NK3B31_group | 0.61172161 | 0.00089047 | positive |
| 4-Methylpentanoylcarnitine | Sphingomonas | 0.61172161 | 0.00089047 | positive |
| Maniladiol | Prevotellaceae_NK3B31_group | -0.61050061 | 0.00091682 | negtive |
| "L-Menthyl (R,S)-3-hydroxybutyrate" | Escherichia-Shigella | 0.60989011 | 0.00093025 | positive |
| Ketotrexate | Escherichia-Shigella | -0.60989011 | 0.00093025 | negtive |
| Beta-Thujaplicin | Prevotellaceae_NK3B31_group | 0.60805861 | 0.00097155 | positive |
| Chalepin acetate | Escherichia-Shigella | -0.60622711 | 0.00101444 | negtive |
| "3-[[5-Methyl-2-(1-methylethyl)cyclohexyl]oxy]-1,2-propanediol" | Muribaculaceae | -0.60509847 | 0.00082624 | negtive |
| Methyl prednisolonate | Muribaculaceae | 0.60387728 | 0.00085207 | positive |
| N-Linoleoyl Tyrosine | Prevotellaceae_NK3B31_group | -0.6037851 | 0.00107416 | negtive |
| SENECIPHYLLINE | Rikenellaceae_RC9_gut_group | 0.60048223 | 0.00092759 | positive |
| "(3R)-3,5-dihydroxy-3-methylpentanoylcarnitine" | Muribaculaceae | 0.60021371 | 0.00093381 | positive |
| Heliangin | Dubosiella | 0.60012299 | 0.00093591 | positive |
| "Nona-5,7-dienedioylcarnitine" | Sphingomonas | 0.5989011 | 0.00120277 | positive |
| Undecylenic acid | Prevotellaceae_NK3B31_group | -0.5989011 | 0.00120277 | negtive |
| 4-Methyl-umbelliferyl-N-acetyl-chitobiose | Escherichia-Shigella | -0.5976801 | 0.00123693 | negtive |
| Eugenol | Rikenellaceae_RC9_gut_group | 0.59703658 | 0.00101012 | positive |
| Caryophyllen-beta | Muribaculaceae | 0.59685545 | 0.00101463 | positive |
| Sarpogrelate | Escherichia-Shigella | -0.5952381 | 0.00130776 | negtive |
| SENECIPHYLLINE | Sphingomonas | 0.59340659 | 0.00136316 | positive |
| Eugenol | Dubosiella | 0.59309149 | 0.00111236 | positive |
| 4-Amino-1-(6-aminopurin-9-yl)pyrimidin-2-one | Rikenellaceae_RC9_gut_group | 0.59202473 | 0.0011415 | positive |
| N-Linoleoyl Tyrosine | Muribaculaceae | -0.59105481 | 0.00116857 | negtive |
| Ketotrexate | Prevotellaceae_NK3B31_group | 0.59035409 | 0.00146 | positive |
| Maniladiol | Escherichia-Shigella | 0.58852259 | 0.0015209 | positive |
| 3'-Hydroxy-T2-triol | Escherichia-Shigella | -0.58791209 | 0.00154169 | negtive |
| Heliangin | Escherichia-Shigella | -0.58730159 | 0.00156272 | negtive |
| Scopolamine | Rikenellaceae_RC9_gut_group | 0.58701289 | 0.00128743 | positive |
| "Nona-5,7-dienedioylcarnitine" | Muribaculaceae | 0.58525417 | 0.00134233 | positive |
| Val-Trp-OH | Escherichia-Shigella | -0.58424908 | 0.00167161 | negtive |
| PA(10:0/a-17:0) | Escherichia-Shigella | -0.58424908 | 0.00167161 | negtive |
| (R)-Laudanidine | Escherichia-Shigella | -0.58180708 | 0.00176337 | negtive |
| (+)-Galeon | Rikenellaceae_RC9_gut_group | 0.58043483 | 0.00150328 | positive |
| "5,7-Megastigmadien-9-ol glucoside" | Prevotellaceae_NK3B31_group | 0.57997558 | 0.00183502 | positive |
| 7C-aglycone | Oscillibacter | 0.57800424 | 0.00159058 | positive |
| Maniladiol | Oscillibacter | -0.57704569 | 0.0016262 | negtive |
| Caryophyllen-beta | Escherichia-Shigella | -0.57692308 | 0.00196 | negtive |
| 4-Methyl-umbelliferyl-N-acetyl-chitobiose | [Eubacterium]_siraeum_group | 0.57688848 | 0.0016321 | positive |
| Heliangin | Rikenellaceae_RC9_gut_group | 0.5763627 | 0.00165199 | positive |
| Goyaglycoside h | Sphingomonas | 0.57448107 | 0.0020652 | positive |
| Beta-Thujaplicin | Dubosiella | 0.571997 | 0.00182535 | positive |
| SENECIPHYLLINE | Dubosiella | 0.57046841 | 0.00188965 | positive |
| SENECIPHYLLINE | Oscillibacter | 0.56873828 | 0.00196477 | positive |
| Arctiopicrin | Escherichia-Shigella | -0.56837607 | 0.00234971 | negtive |
| Arctiopicrin | Prevotellaceae_NK3B31_group | 0.56654457 | 0.00244138 | positive |
| "(3R)-3,5-dihydroxy-3-methylpentanoylcarnitine" | Rikenellaceae_RC9_gut_group | 0.56539928 | 0.00211702 | positive |
| Scopolamine | Oscillibacter | 0.56522361 | 0.00212531 | positive |
| Heliangin | Prevotellaceae_NK3B31_group | 0.56410256 | 0.00256837 | positive |
| 4-Amino-1-(6-aminopurin-9-yl)pyrimidin-2-one | Prevotellaceae_NK3B31_group | 0.56410256 | 0.00256837 | positive |
| Arctiopicrin | Dubosiella | 0.56160261 | 0.00230237 | positive |
| Islatravir | Oscillibacter | 0.55947232 | 0.00241234 | positive |
| Eugenol | Escherichia-Shigella | -0.55921856 | 0.00283947 | negtive |
| "(S,E)-Zearalenone" | Sphingomonas | 0.55799756 | 0.00291096 | positive |
| "Nona-5,7-dienedioylcarnitine" | Prevotellaceae_NK3B31_group | 0.55677656 | 0.00298399 | positive |
| "6-Chloro-5-methyl-N-quinolin-5-yl-2,3-dihydroindole-1-carboxamide" | Dubosiella | 0.55589572 | 0.00260712 | positive |
| Sarpogrelate | Dubosiella | 0.55518255 | 0.00264753 | positive |
| Val-Trp-OH | Rikenellaceae_RC9_gut_group | 0.55506234 | 0.0026544 | positive |
| Sarpogrelate | Rikenellaceae_RC9_gut_group | 0.5547491 | 0.00267235 | positive |
| "(3R)-3,5-dihydroxy-3-methylpentanoylcarnitine" | Escherichia-Shigella | -0.55433455 | 0.00313478 | negtive |
| "Nona-5,7-dienedioylcarnitine" | Rikenellaceae_RC9_gut_group | 0.55412262 | 0.00270858 | positive |
| 4-Methyl-umbelliferyl-N-acetyl-chitobiose | Prevotellaceae_NK3B31_group | 0.55372405 | 0.00317348 | positive |
| Eugenol | Sphingomonas | 0.55250305 | 0.00325212 | positive |
| 5-Pentacosenylresorcinol | Prevotellaceae_NK3B31_group | -0.55189255 | 0.00329206 | negtive |
| "(2S,3R)-3-hydroxy-2-methylpentanedioylcarnitine" | Escherichia-Shigella | -0.54273504 | 0.00394361 | negtive |
| PA(10:0/a-17:0) | Prevotellaceae_NK3B31_group | 0.54029304 | 0.00413493 | positive |
| Pentoprilat | Prevotellaceae_NK3B31_group | 0.53724054 | 0.00438519 | positive |
| (R)-Laudanidine | Oscillibacter | 0.53614766 | 0.00394311 | positive |
| "L-Menthyl (R,S)-3-hydroxybutyrate" | Oscillibacter | -0.5348696 | 0.00404669 | negtive |
| 7C-aglycone | Muribaculaceae | 0.53457488 | 0.0040709 | positive |
| Pentoprilat | Sphingomonas | 0.53418803 | 0.00464828 | positive |
| Beta-Thujaplicin | Sphingomonas | 0.53296703 | 0.00475723 | positive |
| 11-Hydroxyprogesterone 11-glucuronide | [Eubacterium]_siraeum_group | 0.53286519 | 0.0042138 | positive |
| Ketotrexate | Rikenellaceae_RC9_gut_group | 0.53250902 | 0.0042441 | positive |
| Beta-Thujaplicin | Oscillibacter | 0.53231347 | 0.00426081 | positive |
| (R)-Laudanidine | Sphingomonas | 0.53174603 | 0.00486835 | positive |
| Ketotrexate | [Eubacterium]_siraeum_group | 0.5313366 | 0.00434515 | positive |
| Scopolamine | Muribaculaceae | 0.53121661 | 0.0043556 | positive |
| 11-Hydroxyprogesterone 11-glucuronide | Escherichia-Shigella | -0.53113553 | 0.00492473 | negtive |
| Jervine | Escherichia-Shigella | 0.52991453 | 0.00503918 | positive |
| Val-Trp-OH | Dubosiella | 0.52889087 | 0.00456254 | positive |
| 5-Pentacosenylresorcinol | Oscillibacter | -0.52847928 | 0.00460002 | negtive |
| Deoxycholylthreonine | Escherichia-Shigella | 0.52747253 | 0.00527488 | positive |
| "24,25,26,27-Tetranor-23-oxo-hydroxyvitamin D3" | Prevotellaceae_NK3B31_group | -0.52564103 | 0.00545777 | negtive |
| Val-Trp-OH | Prevotellaceae_NK3B31_group | 0.52503053 | 0.00551992 | positive |
| Paramethasone acetate | Sphingomonas | 0.52503053 | 0.00551992 | positive |
| Islatravir | Rikenellaceae_RC9_gut_group | 0.52436477 | 0.00498925 | positive |
| Arctiopicrin | Rikenellaceae_RC9_gut_group | 0.52279856 | 0.00514458 | positive |
| "2-[(3alpha,7alpha,12alpha-Trihydroxy-24-oxocholane-24-yl)amino]ethanesulfonic acid" | Prevotellaceae_NK3B31_group | -0.52136752 | 0.00590562 | negtive |
| 3'-Hydroxy-T2-triol | Sphingomonas | 0.51831502 | 0.00624434 | positive |
| "5,7-Megastigmadien-9-ol glucoside" | [Eubacterium]_siraeum_group | 0.51666217 | 0.00579325 | positive |
| Goyaglycoside h | Dubosiella | 0.51574502 | 0.00589589 | positive |
| 4-Amino-1-(6-aminopurin-9-yl)pyrimidin-2-one | [Eubacterium]_siraeum_group | 0.51574502 | 0.00589589 | positive |
| Alpha-Acetolactate decarboxylase (enzyme preparation from bacillus subtilis recombinant) | Oscillibacter | 0.51505961 | 0.0059736 | positive |
| Arctiopicrin | Sphingomonas | 0.51465201 | 0.00667247 | positive |
| Undecylenic acid | Sphingomonas | -0.51465201 | 0.00667247 | negtive |
| "SM(d18:2(4E,14Z)/22:6(5Z,8E,10Z,13Z,15E,19Z)-2OH(7S, 17S))" | Muribaculaceae | -0.51442528 | 0.00604629 | negtive |
| Alpha-Acetolactate decarboxylase (enzyme preparation from bacillus subtilis recombinant) | Escherichia-Shigella | -0.51282051 | 0.00689571 | negtive |
| Crocin 3 | Lachnospiraceae_UCG-001 | -0.51217116 | 0.00631067 | negtive |
| "Rac-5,6-Epoxy-retinoyl-beta-D-glucuronide" | Prevotellaceae_NK3B31_group | 0.51159951 | 0.00704804 | positive |
| Stallimycin | Prevotellaceae_NK3B31_group | 0.51037851 | 0.00720322 | positive |
| Caryophyllen-beta | Rikenellaceae_RC9_gut_group | 0.51026894 | 0.0065413 | positive |
| Deoxycholylthreonine | Oscillibacter | -0.50962784 | 0.00662061 | negtive |
| "(S,E)-Zearalenone" | Oscillibacter | 0.50962784 | 0.00662061 | positive |
| Strophanthidin | Muribaculaceae | 0.50923524 | 0.00666958 | positive |
| "2-[(3alpha,7alpha,12alpha-Trihydroxy-24-oxocholane-24-yl)amino]ethanesulfonic acid" | Rikenellaceae_RC9_gut_group | -0.50901598 | 0.00669706 | negtive |
| "(2S,3R)-3-hydroxy-2-methylpentanedioylcarnitine" | Rikenellaceae_RC9_gut_group | 0.50901598 | 0.00669706 | positive |
| Maniladiol | Sphingomonas | -0.50671551 | 0.00768624 | negtive |
| (+)-Galeon | Dubosiella | 0.50565635 | 0.00713022 | positive |
| Islatravir | Dubosiella | 0.50412777 | 0.00733497 | positive |
| Methyl prednisolonate | Prevotellaceae_NK3B31_group | 0.5030525 | 0.00819643 | positive |
| N-Linoleoyl Tyrosine | Escherichia-Shigella | 0.502442 | 0.00828419 | positive |
| "3,5-Dimethyl-3'-isopropyl-L-thyronine" | Rikenellaceae_RC9_gut_group | 0.50243792 | 0.00756705 | positive |
| Scopolamine | Escherichia-Shigella | -0.5018315 | 0.00837274 | negtive |
| (R)-Laudanidine | Dubosiella | 0.50137631 | 0.00771599 | positive |
| "L-Menthyl (R,S)-3-hydroxybutyrate" | Sphingomonas | -0.501221 | 0.00846208 | negtive |
| "Nona-5,7-dienedioylcarnitine" | Oscillibacter | 0.4987643 | 0.00809296 | positive |
| Goyaglycoside h | Rikenellaceae_RC9_gut_group | 0.49867904 | 0.00810552 | positive |
| (+)-Galeon | Sphingomonas | 0.4981685 | 0.00892102 | positive |
| Methyl prednisolonate | Escherichia-Shigella | -0.497558 | 0.00901529 | negtive |
| Heliangin | Oscillibacter | 0.49588866 | 0.00852573 | positive |
| Goyaglycoside h | Oscillibacter | 0.49556914 | 0.00857499 | positive |
| "L-Menthyl (R,S)-3-hydroxybutyrate" | Prevotellaceae_NK3B31_group | -0.495116 | 0.00940082 | negtive |
| Zizybeoside II | Muribaculaceae | 0.49397039 | 0.00882508 | positive |
| 4-Amino-1-(6-aminopurin-9-yl)pyrimidin-2-one | Oscillibacter | 0.49237398 | 0.00908086 | positive |
| "24,25,26,27-Tetranor-23-oxo-hydroxyvitamin D3" | Escherichia-Shigella | 0.49206349 | 0.00990221 | positive |
| "3,5-Dimethyl-3'-isopropyl-L-thyronine" | Oscillibacter | 0.49109592 | 0.00929009 | positive |
| Zizybeoside II | Escherichia-Shigella | -0.49084249 | 0.01010899 | negtive |
| Crocin 3 | Prevotellaceae_NK3B31_group | 0.49023199 | 0.01021373 | positive |
| (+)-Galeon | Oscillibacter | 0.48917883 | 0.00961148 | positive |
| "Rac-5,6-Epoxy-retinoyl-beta-D-glucuronide" | Escherichia-Shigella | -0.48778999 | 0.0106419 | negtive |
| Sarpogrelate | Prevotellaceae_NK3B31_group | 0.48778999 | 0.0106419 | positive |
| Methyl prednisolonate | Sphingomonas | 0.48656899 | 0.01086159 | positive |
| 11-Hydroxyprogesterone 11-glucuronide | Oscillibacter | 0.48598367 | 0.01016778 | positive |
| "2-[(3alpha,7alpha,12alpha-Trihydroxy-24-oxocholane-24-yl)amino]ethanesulfonic acid" | Oscillibacter | -0.48406657 | 0.01051428 | negtive |
| Paramethasone acetate | Oscillibacter | 0.48374706 | 0.01057298 | positive |
| "(S,E)-Zearalenone" | [Eubacterium]_siraeum_group | 0.48333899 | 0.01064834 | positive |
| Eugenol | Oscillibacter | 0.48246899 | 0.0108105 | positive |
| "3-[[5-Methyl-2-(1-methylethyl)cyclohexyl]oxy]-1,2-propanediol" | Oscillibacter | -0.47607867 | 0.01206588 | negtive |
| Val-Trp-OH | Sphingomonas | 0.47435897 | 0.01327633 | positive |
| PA(10:0/a-17:0) | Sphingomonas | 0.47435897 | 0.01327633 | positive |
| Chalepin acetate | Sphingomonas | 0.47435897 | 0.01327633 | positive |
| Scopolamine | Dubosiella | 0.47416748 | 0.01246405 | positive |
| 5-Pentacosenylresorcinol | Escherichia-Shigella | 0.47252747 | 0.01367463 | positive |
| "24,25,26,27-Tetranor-23-oxo-hydroxyvitamin D3" | Rikenellaceae_RC9_gut_group | -0.47236683 | 0.01284911 | negtive |
| Heliangin | Sphingomonas | 0.47130647 | 0.01394568 | positive |
| 4-Methyl-umbelliferyl-N-acetyl-chitobiose | Rikenellaceae_RC9_gut_group | 0.4682947 | 0.01375644 | positive |
| Arctiopicrin | Oscillibacter | 0.46809078 | 0.01380323 | positive |
| "3-[[5-Methyl-2-(1-methylethyl)cyclohexyl]oxy]-1,2-propanediol" | Prevotellaceae_NK3B31_group | -0.46764347 | 0.01478595 | negtive |
| Islatravir | Sphingomonas | 0.46703297 | 0.01493002 | positive |
| "5,7-Megastigmadien-9-ol glucoside" | Sphingomonas | 0.46642247 | 0.01507527 | positive |
| "(S,E)-Zearalenone" | Rikenellaceae_RC9_gut_group | 0.46610201 | 0.01426657 | positive |
| "24,25,26,27-Tetranor-23-oxo-hydroxyvitamin D3" | Oscillibacter | -0.46297852 | 0.01502019 | negtive |
| Val-Trp-OH | Oscillibacter | 0.46233949 | 0.01517836 | positive |
| "(2S,3R)-3-hydroxy-2-methylpentanedioylcarnitine" | Dubosiella | 0.4604102 | 0.01566424 | positive |
| Pentoprilat | Muribaculaceae | 0.45947184 | 0.01590516 | positive |
| Caryophyllen-beta | Oscillibacter | 0.45914433 | 0.01598996 | positive |
| (+)-Galeon | [Eubacterium]_siraeum_group | 0.45613016 | 0.01678807 | positive |
| Jervine | Prevotellaceae_NK3B31_group | -0.45543346 | 0.01789828 | negtive |
| "3,5-Dimethyl-3'-isopropyl-L-thyronine" | Dubosiella | 0.45399014 | 0.01737439 | positive |
| "3,5-Dimethyl-3'-isopropyl-L-thyronine" | Sphingomonas | 0.45360195 | 0.01840897 | positive |
| Scopolamine | Prevotellaceae_NK3B31_group | 0.45177045 | 0.01893169 | positive |
| Paramethasone acetate | Muribaculaceae | 0.45153412 | 0.01806794 | positive |
| Deoxycholylthreonine | Prevotellaceae_NK3B31_group | -0.45115995 | 0.01910865 | negtive |
| 5-Pentacosenylresorcinol | Sphingomonas | -0.45054945 | 0.01928697 | negtive |
| 11-Hydroxyprogesterone 11-glucuronide | Prevotellaceae_NK3B31_group | 0.44627595 | 0.02057427 | positive |
| Stallimycin | Sphingomonas | 0.44566545 | 0.02076384 | positive |
| 7C-aglycone | Escherichia-Shigella | -0.44505495 | 0.02095485 | negtive |
| Paramethasone acetate | Dubosiella | 0.4445129 | 0.02017703 | positive |
| "L-Menthyl (R,S)-3-hydroxybutyrate" | Rikenellaceae_RC9_gut_group | -0.44417517 | 0.02028336 | negtive |
| PA(10:0/a-17:0) | Rikenellaceae_RC9_gut_group | 0.44354869 | 0.02048179 | positive |
| Paramethasone acetate | Escherichia-Shigella | -0.44017094 | 0.0225359 | negtive |
| Strophanthidin | Prevotellaceae_NK3B31_group | 0.43772894 | 0.02336258 | positive |
| "Rac-5,6-Epoxy-retinoyl-beta-D-glucuronide" | [Eubacterium]_siraeum_group | 0.43717569 | 0.02259214 | positive |
| Paramethasone acetate | Prevotellaceae_NK3B31_group | 0.43467643 | 0.02443091 | positive |
| Jervine | Oscillibacter | -0.43454161 | 0.02351462 | negtive |
| Alpha-Acetolactate decarboxylase (enzyme preparation from bacillus subtilis recombinant) | Dubosiella | 0.43442423 | 0.02355642 | positive |
| Alpha-Acetolactate decarboxylase (enzyme preparation from bacillus subtilis recombinant) | Sphingomonas | 0.43345543 | 0.02486935 | positive |
| 4-Methylpentanoylcarnitine | Escherichia-Shigella | -0.43345543 | 0.02486935 | negtive |
| "Nona-5,7-dienedioylcarnitine" | Dubosiella | 0.43289564 | 0.02410645 | positive |
| Strophanthidin | [Eubacterium]_siraeum_group | 0.43289564 | 0.02410645 | positive |
| Methyl prednisolonate | Oscillibacter | 0.43134645 | 0.0246745 | positive |
| "3-[[5-Methyl-2-(1-methylethyl)cyclohexyl]oxy]-1,2-propanediol" | Sphingomonas | -0.42796093 | 0.02692315 | negtive |
| 3'-Hydroxy-T2-triol | Lachnospiraceae_UCG-001 | -0.42335535 | 0.0277799 | negtive |
| N-Linoleoyl Tyrosine | Sphingomonas | -0.42002442 | 0.03013341 | negtive |
| "3,5-Dimethyl-3'-isopropyl-L-thyronine" | [Eubacterium]_siraeum_group | 0.41944409 | 0.02941109 | positive |
| 7C-aglycone | Rikenellaceae_RC9_gut_group | 0.41911592 | 0.02955139 | positive |
| Alpha-Acetolactate decarboxylase (enzyme preparation from bacillus subtilis recombinant) | [Eubacterium]_siraeum_group | 0.41730406 | 0.03033577 | positive |
| Islatravir | [Eubacterium]_siraeum_group | 0.41730406 | 0.03033577 | positive |
| Paramethasone acetate | [Eubacterium]_siraeum_group | 0.41638691 | 0.03073916 | positive |
| "3-[[5-Methyl-2-(1-methylethyl)cyclohexyl]oxy]-1,2-propanediol" | Escherichia-Shigella | 0.41636142 | 0.03171701 | positive |
| "SM(d18:2(4E,14Z)/22:6(5Z,8E,10Z,13Z,15E,19Z)-2OH(7S, 17S))" | Sphingomonas | -0.41514042 | 0.03225971 | negtive |
| "2-[(3alpha,7alpha,12alpha-Trihydroxy-24-oxocholane-24-yl)amino]ethanesulfonic acid" | Sphingomonas | -0.41147741 | 0.0339333 | negtive |
| PA(10:0/a-17:0) | Dubosiella | 0.40874398 | 0.03427146 | positive |
| Stallimycin | Muribaculaceae | 0.40787666 | 0.03469205 | positive |
| "(S,E)-Zearalenone" | Dubosiella | 0.40721539 | 0.0350155 | positive |
| N-Linoleoyl Tyrosine | Oscillibacter | -0.40322905 | 0.03701681 | negtive |
| "L-Menthyl (R,S)-3-hydroxybutyrate" | Dubosiella | -0.40110105 | 0.03812196 | negtive |
| Undecylenic acid | Oscillibacter | -0.40099244 | 0.03817906 | negtive |
| "(2S,3R)-3-hydroxy-2-methylpentanedioylcarnitine" | Lachnospiraceae_UCG-001 | -0.40098693 | 0.03818196 | negtive |
| Undecylenic acid | Rikenellaceae_RC9_gut_group | -0.40032149 | 0.03853334 | negtive |
| 3'-Hydroxy-T2-triol | Oscillibacter | 0.40003389 | 0.038686 | positive |
| Methyl prednisolonate | Lachnospiraceae_UCG-001 | -0.39901324 | 0.03923166 | negtive |
| 7C-aglycone | [Eubacterium]_siraeum_group | 0.39896103 | 0.03925974 | positive |
| Jervine | Sphingomonas | -0.3980464 | 0.04068335 | negtive |
| Scopolamine | Sphingomonas | 0.3962149 | 0.04168226 | positive |
| Zizybeoside II | [Eubacterium]_siraeum_group | 0.39529242 | 0.041273 | positive |
| 7C-aglycone | Prevotellaceae_NK3B31_group | 0.39438339 | 0.04270081 | positive |
| Maniladiol | Dubosiella | -0.39437527 | 0.04178891 | negtive |
| Jervine | Rikenellaceae_RC9_gut_group | -0.39405668 | 0.04196932 | negtive |
| "(2S,3R)-3-hydroxy-2-methylpentanedioylcarnitine" | Oscillibacter | 0.39396309 | 0.04202243 | positive |
| Chalepin acetate | Oscillibacter | 0.39268503 | 0.04275311 | positive |
| Heliangin | Lachnospiraceae_UCG-001 | -0.39111851 | 0.0436624 | negtive |
| Sarpogrelate | Oscillibacter | 0.38853132 | 0.04519763 | positive |
| Crocin 3 | Muribaculaceae | 0.38833766 | 0.04531424 | positive |
| Pentoprilat | Escherichia-Shigella | -0.38766789 | 0.04660797 | negtive |
| "Rac-5,6-Epoxy-retinoyl-beta-D-glucuronide" | Oscillibacter | 0.38437761 | 0.04775139 | positive |
| Pentoprilat | Oscillibacter | 0.382141 | 0.04917285 | positive |
| "24,25,26,27-Tetranor-23-oxo-hydroxyvitamin D3" | Sphingomonas | -0.38034188 | 0.05119138 | negtive |
| Chalepin acetate | Dubosiella | 0.37939512 | 0.0509633 | positive |
| 4-Methyl-umbelliferyl-N-acetyl-chitobiose | Oscillibacter | 0.37766778 | 0.05211559 | positive |
| Methyl prednisolonate | Rikenellaceae_RC9_gut_group | 0.37745492 | 0.05225899 | positive |
| Pentoprilat | Dubosiella | 0.37664367 | 0.05280836 | positive |
| Chalepin acetate | Rikenellaceae_RC9_gut_group | 0.37432252 | 0.05440523 | positive |
| "5,7-Megastigmadien-9-ol glucoside" | Rikenellaceae_RC9_gut_group | 0.37400928 | 0.0546236 | positive |
| Maniladiol | Rikenellaceae_RC9_gut_group | -0.3733828 | 0.05506238 | negtive |
| Alpha-Acetolactate decarboxylase (enzyme preparation from bacillus subtilis recombinant) | Rikenellaceae_RC9_gut_group | 0.37275631 | 0.05550391 | positive |
| 5-Pentacosenylresorcinol | Rikenellaceae_RC9_gut_group | -0.36993715 | 0.05752516 | negtive |
| 11-Hydroxyprogesterone 11-glucuronide | Rikenellaceae_RC9_gut_group | 0.36931067 | 0.05798203 | positive |
| 3'-Hydroxy-T2-triol | Rikenellaceae_RC9_gut_group | 0.36805771 | 0.05890424 | positive |
| Arginylmethionine | Lachnospiraceae_UCG-001 | -0.36776324 | 0.05912263 | negtive |
| 4-Methylpentanoylcarnitine | Dubosiella | 0.36716643 | 0.05956717 | positive |
| 3'-Hydroxy-T2-triol | [Eubacterium]_siraeum_group | 0.36563785 | 0.06071763 | positive |
| 4-Methylpentanoylcarnitine | [Eubacterium]_siraeum_group | 0.36441498 | 0.06165037 | positive |
| "(3R)-3,5-dihydroxy-3-methylpentanoylcarnitine" | Dubosiella | 0.36441498 | 0.06165037 | positive |
| Caryophyllen-beta | Dubosiella | 0.36410926 | 0.06188529 | positive |
| Arginylmethionine | Sphingomonas | 0.36385836 | 0.06281816 | positive |
| "Nona-5,7-dienedioylcarnitine" | [Eubacterium]_siraeum_group | 0.36288639 | 0.0628319 | positive |
| Zizybeoside II | Dubosiella | 0.36074637 | 0.06451547 | positive |
| Goyaglycoside h | [Eubacterium]_siraeum_group | 0.3576892 | 0.06698089 | positive |
| Maniladiol | [Eubacterium]_siraeum_group | -0.35707776 | 0.06748259 | negtive |
| 5-Pentacosenylresorcinol | [Eubacterium]_siraeum_group | -0.352492 | 0.07133836 | negtive |
| Crocin 3 | Dubosiella | 0.35188057 | 0.071865 | positive |
| SENECIPHYLLINE | [Eubacterium]_siraeum_group | 0.35035198 | 0.07319468 | positive |
| Deoxycholylthreonine | [Eubacterium]_siraeum_group | -0.34974055 | 0.0737318 | negtive |
| PA(10:0/a-17:0) | Oscillibacter | 0.34507716 | 0.07792848 | positive |
| Sarpogrelate | Sphingomonas | 0.34493284 | 0.07862666 | positive |
| "2-[(3alpha,7alpha,12alpha-Trihydroxy-24-oxocholane-24-yl)amino]ethanesulfonic acid" | Dubosiella | -0.34484907 | 0.07813833 | negtive |
| Eugenol | Lachnospiraceae_UCG-001 | -0.34210534 | 0.08069655 | negtive |
| Strophanthidin | Escherichia-Shigella | -0.33943834 | 0.08374996 | negtive |
| "3-[[5-Methyl-2-(1-methylethyl)cyclohexyl]oxy]-1,2-propanediol" | Rikenellaceae_RC9_gut_group | -0.33892633 | 0.08373986 | negtive |
| PA(10:0/a-17:0) | Lachnospiraceae_UCG-001 | -0.33881586 | 0.08384715 | negtive |
| Ketotrexate | Oscillibacter | 0.3358112 | 0.08680583 | positive |
| Arginylmethionine | Prevotellaceae_NK3B31_group | 0.33516484 | 0.08790976 | positive |
| "2-[(3alpha,7alpha,12alpha-Trihydroxy-24-oxocholane-24-yl)amino]ethanesulfonic acid" | [Eubacterium]_siraeum_group | -0.3347604 | 0.08785898 | negtive |
| 4-Amino-1-(6-aminopurin-9-yl)pyrimidin-2-one | Dubosiella | 0.3347604 | 0.08785898 | positive |
| Deoxycholylthreonine | Sphingomonas | -0.33455433 | 0.08851674 | negtive |
| 4-Amino-1-(6-aminopurin-9-yl)pyrimidin-2-one | Sphingomonas | 0.33394383 | 0.08912694 | positive |
| Deoxycholylthreonine | Rikenellaceae_RC9_gut_group | -0.3317218 | 0.09095884 | negtive |
| Caryophyllen-beta | Lachnospiraceae_UCG-001 | -0.33125007 | 0.09144738 | negtive |
| (R)-Laudanidine | [Eubacterium]_siraeum_group | 0.32956321 | 0.09321056 | positive |
| Strophanthidin | Sphingomonas | 0.32905983 | 0.09412537 | positive |
| Stallimycin | Escherichia-Shigella | -0.32783883 | 0.0954078 | negtive |
| "(3R)-3,5-dihydroxy-3-methylpentanoylcarnitine" | Lachnospiraceae_UCG-001 | -0.32631586 | 0.09667675 | negtive |
| Methyl prednisolonate | [Eubacterium]_siraeum_group | 0.3258946 | 0.09713338 | positive |
| Ketotrexate | Dubosiella | 0.32558889 | 0.09746579 | positive |
| Jervine | [Eubacterium]_siraeum_group | -0.32497745 | 0.09813316 | negtive |
| Ketotrexate | Sphingomonas | 0.32478632 | 0.09867219 | positive |
| Crocin 3 | Oscillibacter | 0.32462814 | 0.09851595 | positive |
| N-Linoleoyl Tyrosine | Rikenellaceae_RC9_gut_group | -0.32357754 | 0.09967402 | negtive |
| "SM(d18:2(4E,14Z)/22:6(5Z,8E,10Z,13Z,15E,19Z)-2OH(7S, 17S))" | Prevotellaceae_NK3B31_group | -0.32356532 | 0.10000146 | negtive |
| "Rac-5,6-Epoxy-retinoyl-beta-D-glucuronide" | Rikenellaceae_RC9_gut_group | 0.32263782 | 0.10071847 | positive |
| "SM(d18:2(4E,14Z)/22:6(5Z,8E,10Z,13Z,15E,19Z)-2OH(7S, 17S))" | Escherichia-Shigella | 0.32112332 | 0.10270079 | positive |
| "(2S,3R)-3-hydroxy-2-methylpentanedioylcarnitine" | [Eubacterium]_siraeum_group | 0.32039169 | 0.10324806 | positive |
| "6-Chloro-5-methyl-N-quinolin-5-yl-2,3-dihydroindole-1-carboxamide" | Lachnospiraceae_UCG-001 | -0.31840247 | 0.10552762 | negtive |
| Crocin 3 | Sphingomonas | 0.31501832 | 0.10969046 | positive |
| Paramethasone acetate | Lachnospiraceae_UCG-001 | -0.31447375 | 0.11013951 | negtive |
| Pentoprilat | Lachnospiraceae_UCG-001 | -0.31381586 | 0.11092618 | negtive |
| Arctiopicrin | Lachnospiraceae_UCG-001 | -0.31282902 | 0.11211399 | negtive |
| Undecylenic acid | Dubosiella | -0.31152589 | 0.11369687 | negtive |
| Stallimycin | Lachnospiraceae_UCG-001 | -0.31151323 | 0.11371233 | negtive |
| (R)-Laudanidine | Lachnospiraceae_UCG-001 | -0.31052638 | 0.1149221 | negtive |
| "(3R)-3,5-dihydroxy-3-methylpentanoylcarnitine" | Oscillibacter | 0.3096109 | 0.11605286 | positive |
| Beta-Thujaplicin | [Eubacterium]_siraeum_group | 0.30938587 | 0.11633205 | positive |
| "3-[[5-Methyl-2-(1-methylethyl)cyclohexyl]oxy]-1,2-propanediol" | [Eubacterium]_siraeum_group | -0.30908015 | 0.11671215 | negtive |
| Caryophyllen-beta | [Eubacterium]_siraeum_group | 0.30755157 | 0.11862638 | positive |
| Undecylenic acid | [Eubacterium]_siraeum_group | -0.30724585 | 0.11901198 | negtive |
| "6-Chloro-5-methyl-N-quinolin-5-yl-2,3-dihydroindole-1-carboxamide" | Rikenellaceae_RC9_gut_group | 0.30695975 | 0.11937367 | positive |
| 5-Pentacosenylresorcinol | Dubosiella | -0.30663442 | 0.11978595 | negtive |
| Arctiopicrin | [Eubacterium]_siraeum_group | 0.30541155 | 0.12134497 | positive |
| "Nona-5,7-dienedioylcarnitine" | Lachnospiraceae_UCG-001 | -0.30427638 | 0.12280546 | negtive |
| Sarpogrelate | Lachnospiraceae_UCG-001 | -0.30394743 | 0.12323107 | negtive |
| Pentoprilat | [Eubacterium]_siraeum_group | 0.30388296 | 0.12331462 | positive |
| "Rac-5,6-Epoxy-retinoyl-beta-D-glucuronide" | Sphingomonas | 0.3028083 | 0.12473635 | positive |
| Goyaglycoside h | Lachnospiraceae_UCG-001 | -0.30032901 | 0.12798441 | negtive |
| "24,25,26,27-Tetranor-23-oxo-hydroxyvitamin D3" | Dubosiella | -0.29868577 | 0.13018667 | negtive |
| Strophanthidin | Oscillibacter | 0.29842784 | 0.13053483 | positive |
| "6-Chloro-5-methyl-N-quinolin-5-yl-2,3-dihydroindole-1-carboxamide" | Muribaculaceae | 0.29657912 | 0.13305017 | positive |
| 7C-aglycone | Dubosiella | 0.29471144 | 0.13562687 | positive |
| "L-Menthyl (R,S)-3-hydroxybutyrate" | [Eubacterium]_siraeum_group | -0.29410001 | 0.13647823 | negtive |
| Sarpogrelate | [Eubacterium]_siraeum_group | 0.28951425 | 0.14298709 | positive |
| 4-Methyl-umbelliferyl-N-acetyl-chitobiose | Dubosiella | 0.28920853 | 0.14342882 | positive |
| Heliangin | [Eubacterium]_siraeum_group | 0.28737423 | 0.14609985 | positive |
| "6-Chloro-5-methyl-N-quinolin-5-yl-2,3-dihydroindole-1-carboxamide" | Sphingomonas | 0.2858452 | 0.1483535 | positive |
| 11-Hydroxyprogesterone 11-glucuronide | Sphingomonas | 0.28449328 | 0.15010638 | positive |
| SENECIPHYLLINE | Lachnospiraceae_UCG-001 | -0.28157901 | 0.15477299 | negtive |
| "(3R)-3,5-dihydroxy-3-methylpentanoylcarnitine" | [Eubacterium]_siraeum_group | 0.28125988 | 0.15526102 | positive |
| Deoxycholylthreonine | Dubosiella | -0.28095416 | 0.15572958 | negtive |
| Chalepin acetate | [Eubacterium]_siraeum_group | 0.2797313 | 0.15761386 | positive |
| "SM(d18:2(4E,14Z)/22:6(5Z,8E,10Z,13Z,15E,19Z)-2OH(7S, 17S))" | Rikenellaceae_RC9_gut_group | -0.27941062 | 0.15811066 | negtive |
| "3-[[5-Methyl-2-(1-methylethyl)cyclohexyl]oxy]-1,2-propanediol" | Dubosiella | -0.27911986 | 0.15856206 | negtive |
| "Rac-5,6-Epoxy-retinoyl-beta-D-glucuronide" | Lachnospiraceae_UCG-001 | -0.27302637 | 0.16823379 | negtive |
| "5,7-Megastigmadien-9-ol glucoside" | Dubosiella | 0.27178265 | 0.17025783 | positive |
| Beta-Thujaplicin | Lachnospiraceae_UCG-001 | -0.26513164 | 0.18137252 | negtive |
| Val-Trp-OH | Lachnospiraceae_UCG-001 | -0.26184216 | 0.18705237 | negtive |
| 11-Hydroxyprogesterone 11-glucuronide | Dubosiella | 0.25833109 | 0.19324958 | positive |
| 7C-aglycone | Sphingomonas | 0.25824176 | 0.19272531 | positive |
| Zizybeoside II | Oscillibacter | 0.25497368 | 0.19930656 | positive |
| Stallimycin | Rikenellaceae_RC9_gut_group | 0.2515322 | 0.205649 | positive |
| N-Linoleoyl Tyrosine | [Eubacterium]_siraeum_group | -0.25099387 | 0.20665342 | negtive |
| Arginylmethionine | Muribaculaceae | 0.24759579 | 0.21307067 | positive |
| Eugenol | [Eubacterium]_siraeum_group | 0.24732527 | 0.21358729 | positive |
| Ketotrexate | Lachnospiraceae_UCG-001 | -0.24572374 | 0.21666309 | negtive |
| 4-Methylpentanoylcarnitine | Lachnospiraceae_UCG-001 | -0.24506584 | 0.21793522 | negtive |
| Val-Trp-OH | [Eubacterium]_siraeum_group | 0.23662516 | 0.23470416 | positive |
| Stallimycin | Dubosiella | 0.23509658 | 0.23783018 | positive |
| 4-Methyl-umbelliferyl-N-acetyl-chitobiose | Lachnospiraceae_UCG-001 | -0.23190794 | 0.24443952 | negtive |
| "24,25,26,27-Tetranor-23-oxo-hydroxyvitamin D3" | [Eubacterium]_siraeum_group | -0.23081653 | 0.2467293 | negtive |
| N-Linoleoyl Tyrosine | Dubosiella | -0.22072786 | 0.26856218 | negtive |
| Chalepin acetate | Lachnospiraceae_UCG-001 | -0.22072373 | 0.26857137 | negtive |
| "6-Chloro-5-methyl-N-quinolin-5-yl-2,3-dihydroindole-1-carboxamide" | Escherichia-Shigella | -0.21988092 | 0.27044993 | negtive |
| 4-Methyl-umbelliferyl-N-acetyl-chitobiose | Sphingomonas | 0.21978022 | 0.26942094 | positive |
| 4-Methylpentanoylcarnitine | Rikenellaceae_RC9_gut_group | 0.21175065 | 0.28900496 | positive |
| (+)-Galeon | Lachnospiraceae_UCG-001 | -0.20822373 | 0.29729841 | negtive |
| Stallimycin | [Eubacterium]_siraeum_group | 0.20697059 | 0.30028071 | positive |
| Strophanthidin | Dubosiella | 0.20697059 | 0.30028071 | positive |
| 4-Methylpentanoylcarnitine | Oscillibacter | 0.20161453 | 0.31323743 | positive |
| Alpha-Acetolactate decarboxylase (enzyme preparation from bacillus subtilis recombinant) | Lachnospiraceae_UCG-001 | -0.1996711 | 0.31802281 | negtive |
| "(S,E)-Zearalenone" | Lachnospiraceae_UCG-001 | -0.19835531 | 0.3212881 | negtive |
| Zizybeoside II | Prevotellaceae_NK3B31_group | 0.1971917 | 0.32268548 | positive |
| "Rac-5,6-Epoxy-retinoyl-beta-D-glucuronide" | Dubosiella | 0.1947419 | 0.33036043 | positive |
| "SM(d18:2(4E,14Z)/22:6(5Z,8E,10Z,13Z,15E,19Z)-2OH(7S, 17S))" | [Eubacterium]_siraeum_group | -0.19382474 | 0.33268767 | negtive |
| "6-Chloro-5-methyl-N-quinolin-5-yl-2,3-dihydroindole-1-carboxamide" | Prevotellaceae_NK3B31_group | 0.19178503 | 0.33789889 | positive |
| Strophanthidin | Lachnospiraceae_UCG-001 | -0.18815794 | 0.34728638 | negtive |
| Scopolamine | Lachnospiraceae_UCG-001 | -0.18717109 | 0.34986717 | negtive |
| Zizybeoside II | Rikenellaceae_RC9_gut_group | 0.18261927 | 0.36191823 | positive |
| Jervine | Dubosiella | -0.18098462 | 0.36630484 | negtive |
| Scopolamine | [Eubacterium]_siraeum_group | 0.17976175 | 0.36960668 | positive |
| Islatravir | Lachnospiraceae_UCG-001 | -0.17894741 | 0.37181507 | negtive |
| Arginylmethionine | Dubosiella | 0.17120167 | 0.39320178 | positive |
| "2-[(3alpha,7alpha,12alpha-Trihydroxy-24-oxocholane-24-yl)amino]ethanesulfonic acid" | Lachnospiraceae_UCG-001 | 0.17039477 | 0.39546914 | positive |
| Deoxycholylthreonine | Lachnospiraceae_UCG-001 | 0.16743425 | 0.40385143 | positive |
| Zizybeoside II | Lachnospiraceae_UCG-001 | -0.16546056 | 0.40949462 | negtive |
| Arginylmethionine | Oscillibacter | 0.16327262 | 0.41580154 | positive |
| Paramethasone acetate | Rikenellaceae_RC9_gut_group | 0.16037919 | 0.42422413 | positive |
| Zizybeoside II | Sphingomonas | 0.15750916 | 0.4309659 | positive |
| 11-Hydroxyprogesterone 11-glucuronide | Lachnospiraceae_UCG-001 | -0.15723688 | 0.43347626 | negtive |
| "3,5-Dimethyl-3'-isopropyl-L-thyronine" | Lachnospiraceae_UCG-001 | -0.1546053 | 0.44130807 | negtive |
| Crocin 3 | Escherichia-Shigella | -0.14957265 | 0.45479777 | negtive |
| "SM(d18:2(4E,14Z)/22:6(5Z,8E,10Z,13Z,15E,19Z)-2OH(7S, 17S))" | Lachnospiraceae_UCG-001 | 0.14703951 | 0.46424255 | positive |
| PA(10:0/a-17:0) | [Eubacterium]_siraeum_group | 0.14093566 | 0.48318905 | positive |
| "6-Chloro-5-methyl-N-quinolin-5-yl-2,3-dihydroindole-1-carboxamide" | [Eubacterium]_siraeum_group | 0.13427137 | 0.50431598 | positive |
| Stallimycin | Oscillibacter | 0.12972345 | 0.51899139 | positive |
| "SM(d18:2(4E,14Z)/22:6(5Z,8E,10Z,13Z,15E,19Z)-2OH(7S, 17S))" | Oscillibacter | -0.12620878 | 0.53047261 | negtive |
| Crocin 3 | [Eubacterium]_siraeum_group | 0.12442693 | 0.53633914 | positive |
| N-Linoleoyl Tyrosine | Lachnospiraceae_UCG-001 | 0.12138161 | 0.54643587 | positive |
| Arginylmethionine | Escherichia-Shigella | -0.11477411 | 0.5671777 | negtive |
| 4-Amino-1-(6-aminopurin-9-yl)pyrimidin-2-one | Lachnospiraceae_UCG-001 | -0.11447371 | 0.56966087 | negtive |
| "6-Chloro-5-methyl-N-quinolin-5-yl-2,3-dihydroindole-1-carboxamide" | Oscillibacter | 0.11284079 | 0.57521474 | positive |
| Jervine | Lachnospiraceae_UCG-001 | 0.11282897 | 0.57525501 | positive |
| "24,25,26,27-Tetranor-23-oxo-hydroxyvitamin D3" | Lachnospiraceae_UCG-001 | 0.11085529 | 0.58199995 | positive |
| 5-Pentacosenylresorcinol | Lachnospiraceae_UCG-001 | 0.10986844 | 0.58538536 | positive |
| Undecylenic acid | Lachnospiraceae_UCG-001 | 0.10131581 | 0.61507781 | positive |
| "3-[[5-Methyl-2-(1-methylethyl)cyclohexyl]oxy]-1,2-propanediol" | Lachnospiraceae_UCG-001 | 0.09605265 | 0.63365281 | positive |
| Pentoprilat | Rikenellaceae_RC9_gut_group | 0.09240598 | 0.64665202 | positive |
| 7C-aglycone | Lachnospiraceae_UCG-001 | -0.09177634 | 0.6489069 | negtive |
| Maniladiol | Lachnospiraceae_UCG-001 | 0.08717107 | 0.66549023 | positive |
| "L-Menthyl (R,S)-3-hydroxybutyrate" | Lachnospiraceae_UCG-001 | 0.08453949 | 0.67503646 | positive |
| Arginylmethionine | Rikenellaceae_RC9_gut_group | 0.08081607 | 0.68862735 | positive |
| Crocin 3 | Rikenellaceae_RC9_gut_group | 0.07799691 | 0.69898103 | positive |
| Arginylmethionine | [Eubacterium]_siraeum_group | 0.06695208 | 0.7400373 | positive |
| "5,7-Megastigmadien-9-ol glucoside" | Lachnospiraceae_UCG-001 | -0.02697369 | 0.89375746 | negtive |
| Strophanthidin | Rikenellaceae_RC9_gut_group | -0.01315611 | 0.94807129 | negtive |
| "SM(d18:2(4E,14Z)/22:6(5Z,8E,10Z,13Z,15E,19Z)-2OH(7S, 17S))" | Dubosiella | -0.01222869 | 0.95172755 | negtive |

**Table S4.** Table of parameters for ROC analysis

| Predictive biomarker | AUC | SE | P value | 95% CI |
| --- | --- | --- | --- | --- |
| Muribaculum | 0.878 | 0.072 | 0.001 | (0.738,1) |
| PA(10:0/a-17:0) | 0.872 | 0.074 | 0.001 | (0.727,1) |
| CA199 | 0.792 | 0.099 | 0.01 | (0.597,0.987) |
| Muri+PA(10:0/a-17:0)+CA199 | 0.917 | 0.057 | <0.001 | (0.805,1) |

SE: Standard error; CI: Confidence intervals

**Table S5.** Spearman correlation analysis parameters table.

| Indicator |  | Ratio | PA (10:0/a-17:0) |
| --- | --- | --- | --- |
| Ratio | Rho | 1 | 0.758* |
|  | P value |  | 0.011 |
| PA (10:0/a-17:0) | Rho | 0.758* | 1 |
|  | P value | 0.011 |  |

*At the 0.05 significance level (two-tailed), the correlation is significant.
